# Supplementary material for: Informal caregiver support needs and burden: a survey in Lithuania
Source: BMJ Open. 2022 Jan 4;12(1):e054607. doi: 10.1136/bmjopen-2021-054607 (PMC8728458; doi:10.1136/bmjopen-2021-054607)
Supplement: Author's manuscript [file bmjopen-2021-054607.draft_revisions.pdf]

BMJ Open is committed to open peer review. As part of this commitment we make the peer review history of every article we publish publicly available.

When an article is published we post the peer reviewers' comments and the authors' responses online. We also post the versions of the paper that were used during peer review. These are the versions that the peer review comments apply to.

The versions of the paper that follow are the versions that were submitted during the peer review process. They are not the versions of record or the final published versions. They should not be cited or distributed as the published version of this manuscript.

BMJ Open is an open access journal and the full, final, typeset and author-corrected version of record of the manuscript is available on our site with no access controls, subscription charges or pay-per-view fees (http://bmjopen.bmj.com).

If you have any questions on BMJ Open's open peer review process please email info.bmjopen@bmj.com

BMJ Open

Informal caregiver support needs and burden: a survey in Lithuania

Journal:	*BMJ Open*
Manuscript ID	bmjopen-2021-054607
Article Type:	Original research
Date Submitted by the Author:	17-Jun-2021
Complete List of Authors:	Biliunaite, Ieva; Linköping University, Department of Behavioural Sciences and Learning<br>Kazlauskas, Evaldas ; Vilnius University, Institute of Psychology<br>Sanderman, Robbert; University of Groningen, Department of Health Psychology; University of Twente, Department of Psychology, Health and Technology<br>Andersson, Gerhard; Linköping University, Department of Behavioural Sciences and Learning; Linköping University, Department of Biomedical and Clinical Sciences
Keywords:	Adult palliative care < PALLIATIVE CARE, PUBLIC HEALTH, COVID-19, International health services < HEALTH SERVICES ADMINISTRATION & MANAGEMENT, Health policy < HEALTH SERVICES ADMINISTRATION & MANAGEMENT, Quality in health care < HEALTH SERVICES ADMINISTRATION & MANAGEMENT

SCHOLARONE™ Manuscripts

I, the Submitting Author has the right to grant and does grant on behalf of all authors of the Work (as defined in the below author licence), an exclusive licence and/or a non-exclusive licence for contributions from authors who are: i) UK Crown employees; ii) where BMJ has agreed a CC-BY licence shall apply, and/or iii) in accordance with the terms applicable for US Federal Government officers or employees acting as part of their official duties; on a worldwide, perpetual, irrevocable, royalty-free basis to BMJ Publishing Group Ltd ("BMJ") its licensees and where the relevant Journal is co-owned by BMJ to the co-owners of the Journal, to publish the Work in this journal and any other BMJ products and to exploit all rights, as set out in our licence.

The Submitting Author accepts and understands that any supply made under these terms is made by BMJ to the Submitting Author unless you are acting as an employee on behalf of your employer or a postgraduate student of an affiliated institution which is paying any applicable article publishing charge ("APC") for Open Access articles. Where the Submitting Author wishes to make the Work available on an Open Access basis (and intends to pay the relevant APC), the terms of reuse of such Open Access shall be governed by a Creative Commons licence – details of these licences and which Creative Commons licence will apply to this Work are set out in our licence referred to above.

Other than as permitted in any relevant BMJ Author's Self Archiving Policies, I confirm this Work has not been accepted for publication elsewhere, is not being considered for publication elsewhere and does not duplicate material already published. I confirm all authors consent to publication of this Work and authorise the granting of this licence.

For peer review only

Informal caregiver support needs and burden: a survey in Lithuania

Ieva Biliunaite[1], MSc; Evaldas Kazlauskas[2], Robbert Sanderman[3,4], Gerhard Andersson[1,5,6]

[1]Department of Behavioural Sciences and Learning, Linköping University, Linköping, Sweden
[2]Center for Psychotraumatology, Institute of Psychology, Vilnius University, Vilnius, Lithuania
[3]Department of Health Psychology, University Medical Center Groningen, University of Groningen, Groningen, Netherlands
[4]Department of Psychology, Health & Technology, University of Twente, Enschede, Netherlands
[5]Department of Biomedical and Clinical Sciences, Linköping University, Linköping, Sweden
[6]Department of Clinical Neuroscience, Karolinska Institutet, Stockholm, Sweden

Corresponding author:
Ieva Biliunaite
Doctoral student
Department of Psychology (PSY)
Department of Behavioural Sciences and Learning,
Linköping University
SE-581 83 Linköping
Sweden
e-mail: ieva.biliunaite@liu.se

For peer review only

ABSTRACT

Objectives: The main objective of this study was to investigate Lithuanian informal caregiver characteristics, support needs and burden. In addition, the impact of the COVID-19 on the caregiver's and care-receiver's well-being was investigated.

Methods: The study was conducted online between May and September 2020. Informal caregivers and individuals with informal caregiving experiences were invited to participate in the survey. The survey questionnaire comprised 38 multiple-choice items including participant demographic characteristics, availability of the support, support needs, well-being, and the impact of the COVID-19 pandemic. In addition, caregiver burden was assessed with the 24-item Caregiver Burden Inventory (CBI).

Results: A total of 226 individuals completed the survey. Most of the participants were women (87.6%). Almost half of the participants (48.7%) were not receiving any support, and a total of 73.9% expressed a need to receive more professional support. Participants were found to experience high burden on the CBI (M=50.21, SD=15.63). Females were found to be significantly more burdened than males (p=.011). Even though many participants experienced psychological problems (55.8%), only 2.2% were receiving any psychological support. Finally, majority of the participants did not experience any changes in their own (63.7%) or the well-being of their care receiver (68.1%) due to the COVID-19 pandemic.

Conclusion: Most of the participants were identified as intensive caregivers experiencing high burden. A majority did not experience changes in their well-being due to the COVID-19. We propose several recommendations for increasing accessibility and availability of support for informal caregivers in Lithuania based on the study findings.

Keywords: adult palliative care; public health; COVID-19; international health services; health policy; quality in health care

Reporting: This report follows the STROBE checklist for cross-sectional studies

Strengths and limitations of this study

- Survey results inform about informal caregiver support needs that extend beyond Lithuania, to neighbour Baltic countries and beyond that share many cultural and historical characteristics.
- Results of this survey encourages further research into the policy and labour market in support for informal caregivers and in attempt to meet the growth of the demand for informal care in the future.
- COVID-19 related items included in the survey provided with valuable insights into the changes of the informal caregiver and care-receiver well-being and needs during the pandemic.
- Taking part in the survey was fully voluntary so it is possible that the sample is not fully representative.
- Limitations of the self-report data should be considered in interpreting the survey's findings.

For peer review only - http://bmjopen.bmj.com/site/about/guidelines.xhtml

For peer review only

INTRODUCTION

Informal caregivers are individuals, who despite no training or experience, provide care for significant others such as partners, children, siblings, parents, or friends. In Europe, the proportion of individuals involved in some form of informal care ranges between 20% to 44%.[1] Due to the increases in the longevity and demand for long-term care, and the limited resources for formal care, it is likely that more people will need to be involved in informal caregiving in the future.[2] It is evident that informal caregivers are not only important for the management of the long-term care, but that they also carry a substantial economic cost[3] and hence, form a backbone of the health and societal care delivery worldwide.[4]

Informal caregiving experience can vary greatly based on several factors. For example, the motivation to provide care,[5] intensity of the caregiving,[6] caregivers skills,[7] and the symptoms of the care-receiver are likely to influence the care.[8] It is known that caregiving can lead to positive experiences, such as personal growth or feelings of closeness and intimacy with the care-receiver.[9] At the same time caregivers also experience worse psychological[10] and physical health[11] than non-carers. In addition, they are at risk of loneliness and social isolation,[12] as well as financial difficulties.[13] Accumulation of these negative outcomes can increase caregiver burden, an experience that is described as a combination of the psychological, physical, social, and financial strains.[4] This concerns many women, as they make-up most informal caregivers,[2] and tend to experience worse negative mental health outcomes than male informal caregivers[4]. Altogether, caregiving could be described as a complex experience that often places caregivers at risk of worse psychological health. Providing caregivers with effective support could help to not only alleviate negative outcomes and improve their quality of life, but also, the quality of care for the car-receiver.[14]

Several studies have been conducted over the last years for investigating informal caregiver support needs. Some of the more commonly observed needs are the need for information and education in the care provision,[15] a need for better collaboration with health-care professionals,[16] flexible work arrangements,[17] a need for social recognition[18] , as well as the availability of professional support.[16] Despite the commonalties of caregiver needs, research show variation in the caregiver well-being across countries.[19] That is, caregiver needs vary based on the country of their location and the specific cultural and socioeconomic background. For this reason, research on country-specific needs of informal caregivers is needed that consider demographic, cultural, and economical influences.[18]

In this study we focused on Lithuanian informal caregivers. As in many other European countries, in Lithuania demand for the informal care is increasing, while the availability of such care is decreasing. More so, this problem is even more evident in Lithuania, were due to mass emigration and one of the fastest aging populations in Europe, informal care resources are shrinking rapidly.[20] The available literature regarding Lithuanian informal caregivers' needs is very limited and is mostly based on small scale, qualitative findings.[21 22] Some of the needs that were outlined were the need for support regarding care-receiver's as well as own well-being,[22] opportunity for formalized training,[23] flexible working conditions[20] and more professional support and respite services.[24] Although it is evident that Lithuanian informal caregivers experience certain strains, more data is needed for gaining knowledge about their basic characteristics and evaluating their challenges so that the following guidelines regarding suitable support options could be proposed. More so, in the light of the COVID-19, as informal caregivers were already identified as a vulnerable group, experiencing more difficulty in providing care and increase in the burden because of the pandemic measures.[25]

For peer review only - http://bmjopen.bmj.com/site/about/guidelines.xhtml

Altogether, the main aim of this research article was to conduct an online survey investigating Lithuanian informal caregiver characteristics, burden, and general support needs as well as the effects of the COVID-19 on the caregiver and care-receiver well-being. The results of this survey will be used for warranting healthcare professional, researcher, policy maker and general public's attention towards to the Lithuanian informal caregiver support needs.

METHOD

Design and sample

An online survey was designed to explore characteristics, experiences, and support needs of Lithuanian informal caregivers. To be eligible, participants had to be informal caregivers or have informal caregiving related experience. Also, they needed to be older than 18 years, fluent in Lithuanian language and have internet access via computer, a mobile phone, or a compatible device. We have followed The Strengthening the Reporting of Observational Studies in Epidemiology (STROBE) Statement: guidelines for reporting observational studies.

Participant and Public Involvement

The development of this survey was inspired by the knowledge obtained following the randomized controlled trial as well as follow-up qualitative interviews with the informal caregivers. Participants and/or the public were not directly involved in the design, or conduct, or reporting or dissemination plans of this research.

Survey development

The survey was conducted in the Lithuanian language. The survey was developed by the authors including a researcher at Vilnius University, with survey items informed by the research questions as well as the current literature. An established 24-item questionnaire measuring caregiver perceived burden, the Caregiver Burden Inventory (CBI),[26] was also included. The final survey consisted of 62 multiple choice questions. Several items also had an option for free-text answers. A short description of the survey items follows below.

Demographic characteristics and caregiving specifics

There were 14 questions dealing with participant demographic characteristics, such as gender and education. In addition, there were 12 questions in relation to the care-receiver and caregiving in general (e.g., care-recipients age and gender; relation to care-recipient; duration of caregiving).

Caregiving knowledge and support needs

In this section participants were presented with two questions in relation to their knowledge and five questions in relation to their needs and currently available as well as preferred support options (e.g., *What are the main caregiving related challenges that you experience;* and *Are you receiving caregiving related support?).*

Caregiver burden

In addition, participants were asked to fill in the CBI questionnaire. The CBI consists of five subscales: Physical health, Emotional health, Time Dependency, Development and Social Relationships. Each of the subscales contain five questions with and exception of the Physical Health, which has four questions. Response options range from 0 ('Never') to 4 ('Nearly Always'). A total score is calculated by summing responses (range 0 to 96), with

For peer review only - http://bmjopen.bmj.com/site/about/guidelines.xhtml

higher total scores indicating higher burden. Sum scores can also be calculated for the subscales separately for Physical Health (range 0 to 16) and for the remaining four subscales (range 0 to 20). Overall, a score of 24 is considered to indicate a need for respite, while scores above 36 - a risk of a burnout.[26] CBI has previously show high reliability.[27] In this sample, reliability coefficients (Cronbach's alpha) were also found to be high: Time Dependency (α= .92), Development (α= .88), Physical Health (α= .87), Emotional Health (α= .84) and Social Relationships (α= .82).

Well-being and support during COVID-19

At the end of the survey, participants were presented with three questions in relation to the COVID-19 pandemic. More specifically, participants were asked how: 1) their own well-being; 2) well-being of the care-recipient and 3) availability of the support have or have not changed due to the pandemic. There were five answer options for answering the third question: *I do not know; improved; did not change; worsened; worsened very much or were not available.* Answer options for the first and second question were presented on a 3-point Likert scale (well-being 1-improved; 2-did not changed; 3-worsened). In answering these questions participants were also requested to indicate type of changes they had observed.

Procedure

The survey was conducted online. Data collection took part between the mid of May and the beginning of September 2020. The link to the survey was disseminated via various social media platforms. The link was also sent to some patient care organizations directly. Interested individuals had to click on the survey link and provide informed consent before taking part in the survey.

Ethics approval for the study was granted by the Vilnius University Psychology Research Ethics Committee, 08-07-2019 No.26. Participation in the survey was voluntary and no monetary compensation was provided.

Statistical Analysis

Data were analysed using IBM SPSS Statistics 25. Descriptive statistics were used for summarizing participant demographic and caregiving related characteristics as well as support needs and the COVID-19 questions responses. Independent samples *t*-tests and One-Way Analysis of Variance (ANOVA) were performed for investigating the association between CBI scores and several demographic characteristics. Multiple linear regression was performed for selected demographic characteristics (as predictors) and CBI total score (as dependent variable). Statistical significance was set at $p<0.05$. When possible, free-text answers were categorized.

RESULTS

I Informal caregiver demographic characteristics

A total of 226 individuals completed the survey. There were no missing data entry points as the survey could only be submitted when all questions had been answered. Demographic characteristics are presented in the Table 1. As it is evident form the table, majority of the participants were female (87.6%). Given the small proportion of male participants and previously observed gender differences in caregiving prevalence and outcomes, we will report results of genders separately.

Most of the participants had obtained a university degree (56.2%), were married, or had a partner (69.5%) and were residing in the capital or one of the larger cities in Lithuania (57%).

For peer review only - http://bmjopen.bmj.com/site/about/guidelines.xhtml

One of the more striking observations was in relation to the occupational status: after starting to provide care a substantial proportion of the participants had either started to be working less than full-time (from 10.6% to 17.3%) or had become unemployed (from 16.8% to 38.9%). This difference was to be statistically significant: $t(225)=-8.69$, $p<0.001$. Regarding self-perceived health, more than half of the sample indicated experiencing physical (58.8%) and psychological health (55.8%) problems.

Table 1. Caregiver characteristics

Participant characteristics	Overall	Women	Men
**Number, n (%)**	226	198 (87.6)	28 (12.4)
**Age caregiver (year): M (SD)**	49.7 (12.7)	49.13 (12.95)	53.89 (10.4)
**Residence: n (%)**			
Capital or one of the larger cities	129 (57)	109 (55.1)	20 (71.4)
Small cities or rural areas	97 (43)	89 (44.9)	8 (28.6)
**Highest education level: n (%)**			
Primary education or vocational training	7 (3.1)	6 (3.0)	1 (3.6)
Secondary education or professional qualification	38 (16.8)	32 (16.2)	6 (21.4)
Applied science or similar	54 (23.9)	50 (25.2)	4 (14.3)
University degree	127 (56.2)	110 (55.6)	17 (60.7)
**Marital status: n (%)**			
Single	32 (14.2)	29 (14.6)	3 (10.7)
Married/partner	157 (69.5)	135 (68.2)	22 (78.6)
Divorced/widowed or other	37 (16.3)	34 (17.2)	3 (10.7)
**Family members: n (%)**			
1-2	81 (35.9)	68 (34.4)	13 (46.5)
3-4	118 (52.2)	106 (53.6)	12 (42.8)
4+	27 (11.9)	24 (12)	3 (10.7)
**Occupational status before caregiving: n (%)**			
Employed full time	164 (72.6)	139 (70.2)	25 (89.3)
Employed part time	24 (10.6)	23 (11.6)	1 (3.6)
Unemployed	38 (16.8)	36 (18.2)	2 (7.1)
**Occupational status after starting caregiving: n (%)**			
Employed full time	99 (43.8)	83 (41.9)	16 (57.1)
Employed part time	39 (17.3)	34 (17.2)	5 (17.9)
Unemployed	88 (38.9)	81 (40.9)	7 (25)
**Financial situation: n (%)**			
Cannot afford enough food	42 (18.6)	40 (20.2)	2 (7.1)
Enough for food, but not for bigger purchases (e.g.: TV)	83 (36.7)	70 (35.4)	13 (46.4)
Enough for bigger, but not very big purchases (e.g.: a flat)	92 (40.7)	81 (40.9)	11 (39.4)
Everything is affordable	9 (4.0)	7 (3.5)	2 (7.1)
**Health problems caregiver: n (yes %)**			
Physical health problems	133 (58.8)	118 (59.6)	15 (53.6)
Psychological health problems	126 (55.8)	115 (58.1)	11 (39.3)

For peer review only - http://bmjopen.bmj.com/site/about/guidelines.xhtml

**Self-rated well-being over last four weeks: n (%)**			
Either very good or good	42 (18.6)	35 (17.6)	7 (25)
Neither good nor bad	82 (36.3)	70 (35.4)	12 (42.9)
Not very good or bad	102 (45.1)	93 (47)	9 (32.1)

II Caregiving specifics

Care-receiver's characteristics as well as caregiving intensity-related information are presented in Table 2. Most of the care-receivers were female (68.1%), and the mean age was 76 years (SD=19.85). The care-receiver's age varied, with the youngest being 5 years and oldest 99 years. However, only 5.8% of informal caregivers were providing care for 0-18 years old, and a majority (54%) provided care in the age range of 80–100 years old. Recipients were most commonly providing care for their parent (father or mother) (57.1%). Among the types of relations as categorized from the free-text answers and not mentioned in the table, the most common for the recipients was to be a grandmother (5%). Most of the care-receivers had dementia (22.6%), a previous experience of stroke or myocardial infarction (15%) or needed assistance because of old age (13.7%). Regarding reasons for care provision, two of the most frequent reasons were own initiative (23%) and having no other family member available for care provision (23%). Other common reasons stated in the free-text boxes were the care-receiver requesting care (6.2%) and being the parent of the care-receiver (6.2%).

Almost half of the participants had provided care for more than four years (48.2%), five to seven days per week (79.6%), and either three to seven (32.7%) or more than 12 hours per day (35.4%). In addition, 8% of the participants used free-text answer option to indicate that the care-receiver was fully dependent on their support.

Table 2. Caregiving information

Caregiving related information	Overall	Women	Men
**Gender care-recipient: n (%)**		154 (68.1)	72 (31.9%)
**Age care-recipient (year): n (%)**	71.38 (23.31)	76.27 (19.85)	60.93 (26.65)
**Relation to care-recipient: n (%)**			
Husband/wife/partner	23 (10.2)	13 (6.6)	10 (35.7)
Father/mother	129 (57.1)	116 (58.6)	13 (46.4)
Parent-in-law/uncle/auntie	23 (10.2)	19 (9.6)	4 (14.3)
Brother/sister	5 (2.2)	5 (2.5)	0
Daughter/son	25 (11.1)	25 (12.6)	0
Other	21 (9.3)	20 (10.1)	1 (3.6)
**Main reason for caregiving: n (%)**			
Old age	31 (13.7)	27 (13.6)	4 (14.3)
Dementia	51 (22.6)	45 (22.7)	6 (21.5)
Stroke/ Myocardial infarction	34 (15)	32 (16.3)	2 (7.1)
Amyotrophic lateral sclerosis (ALS)	12 (5.3)	6 (3)	6 (21.5)
Cerebral palsy	11 (4.9)	11 (5.6)	0
Cancer	10 (4.4)	8 (4)	2 (7.1)
Other	77 (34.1)	69 (34.8)	8 (28.5)
**Caregiver resides with care receiver: n (yes %)**	163 (72.1)	142 (71.7)	21 (75)
**Caregiving circumstances: n (single caregiver %)**	92 (40.7)	76 (38.4)	16 (57.1)

For peer review only - http://bmjopen.bmj.com/site/about/guidelines.xhtml

**Reasons for providing care: n (%)**			
Own initiative	52 (23)	44 (22.2)	8 (28.6)
Due to close living proximity to the care-receiver	26 (11.5)	21 (10.6)	5 (17.9)
There were no other family members available for caregiving	52 (23)	46 (23.2)	6 (21.4)
This what was agreed on together with other family members	41 (18.1)	39 (19.7)	2 (7.1)
Other	55 (24.4)	48 (24.3)	7 (25)
**Time caring: n (in months %)**			
<12	47 (20.8)	45 (22.7)	2 (7.1)
12-24	36 (16)	28 (14.1)	8 (28.6)
24-48	34 (15)	32 (16.2)	2 (7.1)
48+	109 (48.2)	93 (47)	16 (57.1)
**Time per week: n (in days %)**			
1-2	26 (11.5)	23 (11.6)	3 (10.7)
3-4	20 (8.8)	17 (8.6)	3 (10.7)
5-7	180 (79.6)	158 (79.8)	22 (78.6)
**Time per day: n (in hours %)**			
3<	51 (22.6)	40 (20.2)	11 (39.3)
3-7	74 (32.7)	64 (32.3)	10 (35.7)
8-11	21 (9.3)	20 (10.1)	1 (3.6)
12+	80 (35.4)	74 (37.4)	6 (21.4)
**Helping care-receiver with basic activities of daily living (ADLs)[a]: n (yes %)**			
Bathing	175 (77.4)	160 (80.8)	15 (53.6)
Brushing teeth	102 (45.1)	95 (48)	7 (25)
Dressing	156 (69)	140 (70.7)	16 (57.1)
Eating	160 (70.8)	143 (72.2)	17 (60.7)
Moving	155 (68.6)	134 (67.7)	21 (75)
Toilet needs	130 (57.5)	116 (58.6)	14 (50)
Maintaining general hygiene (e.g., cutting nails)	189 (83.6)	170 (85.9)	19 (67.9)
**Helping care-receiver with instrumental activities of daily living (IADLs)[a]: n (yes %)**			
Using telephone	100 (44.2)	87 (43.9)	13 (46.4)
Laundry	167 (73.9)	143 (72.2)	24 (85.7)
Shopping	170 (75.2)	146 (73.7)	24 (85.7)
Transportation	158 (69.9)	134 (67.7)	24 (85.7)
Cooking	172 (76.1)	148 (74.7)	24 (85.7)
Medication	164 (72.6)	147 (74.2)	17 (60.7
Household	179 (79.2)	152 (76.8)	27 (96.4)
Financial management	139 (61.5)	122 (61.6)	17 (60.7)

[a]Possible to choose more than one response option.

III Informal caregiver knowledge and support needs

Several aspects in relation to the informal caregivers knowledge and needs were identified (Table 3). Almost half of the participants (47.3%) had no specific knowledge about the disorder of the care-recipient, and more than half (55.3%) had no knowledge about how to provide care in general. Consequently, a majority wished to receive more professional

For peer review only

support in their role as caregivers (73.9%). Less time for oneself and changes in physical and mental health were identified as the two most prominent challenges, 84.5% and 76.5% respectively. Regarding support, most of participants either did (34.5%) or did not look for profession support (35%). In turn, almost half of the participants reported that they were not receiving any caregiving-related support (48.7%). Regarding the ones receiving support, financial aid was the most mentioned (33.6%). Only 2.2% of the participants received psychological help. Participants reported that their situation would improve if caregiving was recognised as part of working experience (55.3%), if they would receive financial (46.5%) or professional support (45.1%), and more days for respite (44.2%).

Table 3. Caregiver's knowledge and support needs

Participant characteristics	Overall	Women	Men
**Number, n (%)**	226	198 (87.6)	28 (12.4)
**Prior care provision knowledge regarding the disorder(s) of care-receiver: n (no knowledge %)**	107 (47.3)	96 (48.5)	11 (39.3)
**Prior care provision knowledge in general: n (no knowledge %)**	125 (55.3)	108 (54.5)	17 (60.7)
**Would you like to receive more professional support with caregiving (medical, social etc): n (yes %)**	167 (73.9)	143 (72.2)	24 (85.7)
**Personal difficulties experienced by caregivers[a]: n (%)**			
Less time for one-self	191 (84.5)	169 (85.4)	22 (78.6)
Changes in sleep quality	128 (56.6)	114 (57.6)	14 (50)
Changes in relationships with other people	122 (54)	110 (55.6)	12 (42.9)
Changes in financial situation	107 (47.3)	95 (48)	12 (42.9)
Changes in physical or psychological health	173 (76.5)	156 (78.8)	17 (60.7)
**Have you searched for caregiving related support[a]: n (%)**			
Have not searched	79 (35)	67 (33.8)	12 (42.9)
Yes, searched financial support	54 (23.9)	45 (22.7)	9 (32.1)
Yes, searched for own well-being support	64 (28.3)	59 (29.8)	5 (17.9)
Yes, looked for professional support for conducting caregiving tasks	78 (34.5)	69 (34.8)	9 (32.1)
**Receiving caregiving related support[a]: n (%)**			
Not receiving support	110 (48.7)	98 (49.5)	12 (42.9)
Receiving financial support	76 (33.6)	68 (34.3)	8 (28.6)
Receiving psychological support	5 (2.2)	5 (2.5)	0
Receiving professional support for caregiving tasks	27 (11.9)	22(11.1)	5 (17.9)
**My situation would improve if[a]: n (%)**			
I would receive psychological support	73 (32.3)	67 (33.8)	6 (21.4)

For peer review only - http://bmjopen.bmj.com/site/about/guidelines.xhtml

I would receive professional caregiving related support	102 (45.1)	88 (44.4)	14 (50)
I would receive more respite days	100 (44.2)	90 (45.5)	10 (35.7)
I would receive financial support	105 (46.5)	87 (43.9)	18 (64.3)
I would receive more information about the care-provision and specific disorder	59 (26.1)	53 (26.8)	10 (35.7)
I would receive more support from people in my close environment	59(26.1)	56 (28.3)	3 (10.7)
If time spent caregiving would add to the years of working	125 (55.3)	109 (55.1)	16 (57.1)
Other			

[a]Possible to choose more than one response option.

IV Informal caregiver burden

Mean scores, standard deviations, and gender differences regarding scores on the CBI and the separate sub-scales are presented in Table 4. Since all the subscales have five items each except for the Physical Health subscale, which has four, the scores for this subscale were multiplied by 1.25.[22] Overall, participants displayed high mean score on the CBI (M=50.21, SD=15.63), with highest mean score on the subscale Time Dependency (M=16.15, SD=4.21). As illustrated in Table 4, female participants scored significantly higher on the overall CBI score (*p*=.011) as well as on the subscales Development (*p*=.035) and Physical Health (*p*=.002).

Table 4. Means, standard deviations and independent samples *t*-test results for CBI total score and separate subscales.

Scale	Mean (SD)			*t*	*p*
	Overall	Female	Male		
CBI[a] total score	50.21 (15.63)	51.2 (15.41)	43.18 (15.68)	-2.57	.011
Time Dependency	16.15 (4.21)	16.30 (4.16)	15.07 (4.48)	-1.45	.149
Development	12.77 (4.85)	13.03 (4.86)	10.96 (4.44)	-2.12	.035
Physical Health	11.07(4.85)	11.44 (4.76)	8.44 (4.75)	-3.12	.002
Emotional Health	4.93 (4.04)	5.05 (4.04)	4.11 (4.0)	-1.15	.251
Social relationships	7.5 (4.71)	7.68 (4.58)	6.29 (5.45)	-1.47	.144

[a]CBI - Caregiver Burden Inventory.

Independent samples *t*-tests or ANOVAs were performed when analysing demographic as well as caregiving-related characteristics (with exception of the care-receiver symptoms, which was not included due to the many categories) in relation to CBI total scores (Table 5). Eight variables (nine if gender is included) were found to be associated with increased CBI scores: physical (*p*<.001) or psychological (*p*<.001) health complaints, poorer self-rated well-being (*p*<.001), residing with the care-receiver (*p*<.001) and caring for longer and with higher

For peer review only - http://bmjopen.bmj.com/site/about/guidelines.xhtml

intensity (*p*<.001). Also, informal caregivers who started providing care as there were no other family members to help were found to be significantly more burdened than individuals who took up this task following own initiative (*p*=.001).

Table 5. Caregiver burden associations with sociodemographic and informal caregiver study variables

Variable	CBI[a], M (SD)	*t* or *F*[b]	*p*
**Age caregiver**			
18-39	47.07 (2.15)	1.61	.203
40-59	51.66 (15.86)		
60-80	48.98 (16.09)		
**Residence**			
Capital or one of the larger cities	48.9 (17.31)	-1.51	.131
Small cities or rural areas	51.95 (12.96)		
**Education**			
Primary education or vocational training	49.71 (15.91)	0.78	.504
Secondary education or professional qualification	48.76 (12.52)		
Applied science or similar	53.02 (14.89)		
University degree	49.47 (16.75)		
**Marital status**			
Single	49.09 (12.57)	0.15	.862
Married/partner	50.21 (15.56)		
Divorced/widowed or other	51.16 (18.44)		
**Family members**			
1-2	49.09 (15.37)	1.93	.148
3-4	51.94 (15.75)		
4+	46.0 (15.33)		
**Financial situation**			
Cannot afford enough food	52.67 (18.82)	0.42	.737
Enough for food, but not for bigger purchases (e.g.: TV)	49.7 (13.60)		
Enough for bigger, but not very big purchases (e.g.: a flat)	49.61 (15.79)		
Everything is affordable	49.56 (16.95)		
**Current occupational status**			
Employed full time	48.52 (16.07)	1.05	.352
Employed part time	51.90 (17.84)		
Unemployed	51.36 (14.01)		
**Physical health problems caregiver**			
No	45.82 (15.74)	-3.59	<.001
Yes	53.28 (14.86)		
**Psychological health problems caregiver**			
No	43.8 (14.83)	-5.86	<.001
Yes	55.29 (14.38)		
**Self-rated well-being over last four weeks**			
Either very good or good	36.88 (15.18)	22.88	<.001
Neither good nor bad	52.09 (12.36)		
Not very good or bad	54.19 (15.35)		
**Gender care-receiver**			
Male	49.04 (15.53)	-0.77	.444
Female	50.75 (16.14)		
**Age care-receiver**			

For peer review only

0-18	53.31 (5.22)	1.14	.337
19-39	54.59 (14.62)		
40-59	44.45 (8.89)		
60-79	50.19 (17.43)		
80-100	50.22 (16.39)		
**Relation care-receiver**			
Husband/wife/partner	45.39 (13.71)	1.72	.132
Father/mother	51.19 (15.87)		
Parent-in-law/uncle/auntie	46.87 (20.09)		
Brother/sister	49.0 (10.56)		
Daughter/son	56.04 (8.24)		
Other	46.48 (16.70)		
**Individual is the only caregiver**			
Yes	52.37 (14.30)	1.73	.085
No	48.72 (16.37)		
**Reason why one started providing care**			
Own initiative	44.17 (16.50)	4.59	.001
Due to the proximity to the care-receiver	47.31 (19.70)		
No other family members available	56.46 (13.17)		
Decided with family members	50.27 (15.32)		
Other	51.33 (12.88)		
**Receiving of caregiving related support**			
Receiving support	48.89 (16.99)	1.31	.191
Not receiving support	51.6 (14.0)		
**Residing with the care-receiver**			
Yes	52.9 (13.28)	8.42	<.001
No	43.24 (18.91)		
**Time Caring: months**			
<12	45.17 (19.20)	7.99	<.001
12-24	42.56 (14.72)		
24-48	51.56 (15.44)		
48+	54.49 (12.62)		
**Time week: days**			
1-2	39.04 (19.87)	8.12	<.001
3-4	49.90 (18.14)		
5-7	51.86 (14.01)		
**Time day: hours**			
<3	39.90 (19.07)	11.82	<.001
3-7	52.12 (13.87)		
8-11	50.0 (9.85)		
12+	55.06 (13.04)		

[a]CBI - Caregiver Burden Inventory.
[b]Either Independent samples t-tests or One-Way Analysis of Variance (ANOVA) were performed dependently on the number of categories.

We ran a hierarchical multiple linear regression including the significant predictors presented in the Table 5. Out of the nine entered predictors, four made a significant independent contribution to CBI total score: self-rated well-being (*p*=.001), caregiving duration in months (*p*=.006), caregiver's gender (*p*=.046), and experience of the psychological health problems (p=.001) (Table 6, Block 1). We ran the regression again with all four variables included. All variables were found to contribute to the model significantly, explaining 27.3% of variance in the CBI scores (Table 6, Block 2).

For peer review only

Table 6. Multiple linear regression results with demographic characteristics as predictors and CBI as an outcome variable

	Block 1				
**Included variables**	**B [95% CI]**	**SE B**	**β**	**p**	**R^2**
Self-rated well-being over last four weeks	4.05 [1.77, 6.32]	1.15	0.23	.001	0.324
Time day: hours	0.94 [-0.78, 2.65]	0.87	0.07	.283	
Time week: days	2.63 [-0.62, 5.89]	1.65	0.11	.112	
Time Caring: months	2.17 [0.64, 3.70]	0.78	0.17	.006	
Reason why one started providing care	0.87 [-0.32, 2.06]	0.60	0.08	.151	
Residing with the care-receiver	2.46 [-2.62, 7.54]	2.58	0.07	.341	
Gender caregiver	5.50 [0.09 10.90]	2.74	0.12	.046	
Physical health problems caregiver	1.43 [-2.46, 5.32]	1.97	0.05	.469	
Psychological health problems caregiver	6.33 [2.48, 10.18]	1.95	0.20	.001	
	**Block 2**				
**Included variables**	**B [95% CI]**	**SE B**	**β**	**p**	**R^2**
Self-rated well-being over last four weeks	4.79 [2.64, 6.94]	1.09	0.27	<.001	0.273
Time Caring: months	3.02 [1.54, 4.50]	0.75	0.23	<.001	
Gender caregiver	6.26 [0.83, 11.69]	2.76	0.13	.024	
Psychological health problems caregiver	6.77 [2.90, 10.63]	1.96	0.22	.001	

V Wellbeing during Covid-19

Out of the 226 participants, a majority indicated that neither their own (63.7%) nor the care-receiver's well-being (68.1%) had changed during the COVID-19 pandemic. Regarding the availability of the care-related support, 27.4% indicated a decrease and 32.3% a very big decrease in the availability of support.

DISCUSSION

The aim of this study was to investigate Lithuanian informal caregiver characteristics, support needs, burden, and the impact of the COVID-19 on the well-being. Overall, Lithuanian informal caregivers displayed high burden, high involvement in the care-provision and limited access to the support options. Most of the participants experienced no changes in their well-being due to the COVID-19. We further discuss the findings below.

Caregiver and caregiving related characteristics

The finding that most of the informal caregivers were females in their 50s is in line with the research literature indicating that most of the informal care in Lithuania, as in other parts of the world, is carried out by middle-aged women.[6] More than half of the participants reported

For peer review only - http://bmjopen.bmj.com/site/about/guidelines.xhtml

either psychological or physical health problems suggesting that caregivers are at risk of poor well-being.[10 11 19] We also observed a significant decrease in the proportion of caregivers working full-time after the start of the care provision. Current labour market-related measures for informal caregivers in Lithuania could be described as limited and insufficient.[20] This might explain why participants in our study had to move from working full-time to either not working at all or working reduced number of hours. Having to reduce work hours due to the caregiving duties alone has previously been found to have a negative effect on the caregiver's psychological well-being.[28] Further efforts to improve current labour market-related measures are most likely be needed to prevent such risks.

Individuals who provide care for 11 or more hours per week have previously been defined as intensive caregivers.[6] In our sample, 77.4% of all the participants fall into this category. Most of these caregivers provided care for five to seven days per week, and at the time of the survey completion, for four or more years. Mental health consequences have previously been found to be even more severe for intensive caregivers,[6] a finding that could at least partly explain the sample's high overall scores on the CBI measure. In line with this, most of the participants indicated that they would like to receive more professional support. In terms of available support, current day care and nursing home services as well as respite services for the informal caregivers in Lithuania could be described as very limited.[20] This suggests that further policy measures for improving both, availability, and accessibility of such services are needed.

Caregiver knowledge and support needs

Most of the participants started providing care without having any general or receiver symptom- specific knowledge about caregiving. Informal caregivers in Lithuania often have to learn about the care provision through own experience.[23] As a consequence, they often experience feelings of anxiety and uncertainty. In addition, almost half of the participants did not receive any support in their caregiving. Among those who had support, a majority received financial support. Time spent for caregiving being counted as work experience was the most favoured suggestion by the caregivers. In addition, a majority indicated that financial and professional support would improve their situation. Interestingly, approximately one third of the participants indicated that they had not searched for support. One explanation could be that they did not know which support is available or how to access it.[23] Prior negative experiences of interactions with health care professionals could also influence health care seeking. A recent qualitative study on Lithuanian informal caregivers reported that some caregivers experienced difficulty in communicating with the healthcare professionals.[21] Studies in other countries have also found that carers experience dissatisfaction with the health care providers in terms of information provision, treatment optimization, involvement of the caregiver and management of caregivers' own health.[29] As a solution, additional training could be offered to the professionals about guiding and supporting informal caregivers.[30] Early initiation of the contact with the caregivers could also be useful. This might be especially important for cases in which help-seeking behaviour conflicts with caregivers' values[31] or caregivers express high needs for continuous or frequent support.

Caregiver burden

In line with the previous literature,[4 6] we found that female participants experienced a higher burden than the males. Participants overall scored the highest on the Time Dependency subscale of the CBI which mirrors a large time investment on caregiving duties. This was further confirmed by the regression analyses, in which being female, longer caregiving duration, poorer self-rated well-being and psychological health problems were significant

For peer review only - http://bmjopen.bmj.com/site/about/guidelines.xhtml

predictors of higher CBI total scores. The question of what type of psychological support options informal caregivers would prefer remains. As identified in the recent qualitative Lithuanian informal caregiver study[23] access to peer support groups as well as internet-based intervention programs could have potential in reducing caregiver psychological burden. Further research into these matters is encouraged.

COVID-19

Contrary to our expectations and recent researcher findings[25 32-34] most of the informal caregivers did not report any changes in own or care-receivers well-being because of the COVID-19 pandemic. Higher appreciation of the life at the start of the pandemic could be one explanation why no changes were observed.[35] As outlined recently, changes in the caregiver burden during the pandemic might be rather complex and vary by gender.[36] Therefore, it is possible that our questions did not capture the complexity of such changes. Future studies are needed to evaluate the impact of the pandemic on informal caregivers.

Study limitations

There are limitations to be addressed. Firstly, our sample might not be representative for Lithuanian caregivers as participants are likely to have higher computer literacy and motivation to participate in online research. Secondly, we only included a few questions about changes in well-being during the COVID-19. More elaborate investigations should be done considering the changing nature of the pandemic. Finally, our study was cross-sectional and did only investigate caregiver needs at a certain point in time. Longitudinal data should be collected for more comprehensive evaluation of the possible fluctuations in well-being and support needs over time.

Conclusion and recommendations

Lithuanian informal caregivers, in relation to caregivers in other European countries, experience high burden and unmet practical as well as psychological support needs. We outline here a few points that could be focused on by policy makers, healthcare professionals and researchers. Firstly, current labour market policies are insufficient in allowing caregivers to balance caregiving, work, and personal life. To prevent possible negative financial and psychological health consequences for the caregivers, further emphasis should be put on adapting current policies. Secondly, we found that the caregivers experienced a need for information and practical support. More accessible information sources and better guidance from health professionals could be offered. Lastly, participants in our study experienced high caregiver burden. Due to the low coverage and accessibility of psychological support options, we encourage researchers to develop innovative support measures, such as online support groups or psychological support interventions.[37]

We conclude that supporting informal caregivers is crucial not only for the individual, but also on a societal level. Meeting these needs is important from the start and throughout the caregiving experience.

For peer review only - http://bmjopen.bmj.com/site/about/guidelines.xhtml

Acknowledgments

We thank George Vlaescu and Austeja Dumarkaite for the help with the study. We also thank all the individuals and organizations that willingly helped with the dissemination of the survey.

Contributors

All authors contributed to the conception and study design of the study. IB and EK contributed to the data collection. IB and GE analysed and interpreted the data. IB drafted the manuscript. EK, RS and GA critically revised the paper. All authors approved the final version of the manuscript.

Funding

This project has received funding from the European Union's Horizon 2020 research and innovation programme under the Marie Skłodowska-Curie grant agreement No 814072 and is part of The European Training Network on Informal Care (ENTWINE).

Competing interests

None declared.

Data availability statement

Anonymized data will be stored at Linköping university for 10 years. It will be available upon reasonable request in excel format after the publication of the manuscript. Primary investigator (Prof Gerhard Andersson, Linköping University; gerhard.andersson@liu.se) should be contacted for requesting about the data.

Ethics approval statement

Ethics approval for the study was granted by the Vilnius University Psychology Research Ethics Committee, 08-07-2019 No.26.

Abbreviations

CBI: Caregiver Burden Inventory

For peer review only

References

1. Verbakel E. How to understand informal caregiving patterns in Europe? The role of formal long-term care provisions and family care norms. *Scandinavian journal of public health* 2018;46(4):436-47.
2. Talley RC, Crews JE. Framing the public health of caregiving. *American Journal of Public Health* 2007;97(2):224-28.
3. Rabarison KM, Bouldin ED, Bish CL, et al. The economic value of informal caregiving for persons with dementia: Results from 38 states, the District of Columbia, and Puerto Rico, 2015 and 2016 BRFSS. *American journal of public health* 2018;108(10):1370-77.
4. Revenson T, Griva K, Luszczynska A, et al. Caregiving in the illness context: Springer 2016.
5. Quinn C, Clare L, Woods RT. The impact of motivations and meanings on the wellbeing of caregivers of people with dementia: a systematic review. *International Psychogeriatrics* 2010;22(1):43.
6. Verbakel E, Tamlagsrønning S, Winstone L, et al. Informal care in Europe: findings from the European Social Survey (2014) special module on the social determinants of health. *The European Journal of Public Health* 2017;27(suppl_1):90-95.
7. Mollica MA, Litzelman K, Rowland JH, et al. The role of medical/nursing skills training in caregiver confidence and burden: a CanCORS study. *Cancer* 2017;123(22):4481-87.
8. Harding R, Gao W, Jackson D, et al. Comparative analysis of informal caregiver burden in advanced cancer, dementia, and acquired brain injury. *Journal of pain and symptom management* 2015;50(4):445-52.
9. Cross AJ, Garip G, Sheffield D. The psychosocial impact of caregiving in dementia and quality of life: a systematic review and meta-synthesis of qualitative research. *Psychology & health* 2018;33(11):1321-42.
10. Sallim AB, Sayampanathan AA, Cuttilan A, et al. Prevalence of mental health disorders among caregivers of patients with Alzheimer disease. *Journal of the American Medical Directors Association* 2015;16(12):1034-41.
11. Berglund E, Lytsy P, Westerling R. Health and wellbeing in informal caregivers and non-caregivers: a comparative cross-sectional study of the Swedish general population. *Health and Quality of Life Outcomes* 2015;13(1):1-11.
12. Vasileiou K, Barnett J, Barreto M, et al. Experiences of loneliness associated with being an informal caregiver: a qualitative investigation. *Frontiers in Psychology* 2017;8:585.
13. Cipollone A, Patacchini E, Vallanti G. Female labour market participation in Europe: novel evidence on trends and shaping factors. *IZA Journal of European Labor Studies* 2014;3(1):1-40.
14. Litzelman K, Kent EE, Mollica M, et al. How does caregiver well-being relate to perceived quality of care in patients with cancer? Exploring associations and pathways. *Journal of Clinical Oncology* 2016;34(29):3554.
15. Docherty A, Owens A, Asadi-Lari M, et al. Knowledge and information needs of informal caregivers in palliative care: a qualitative systematic review. *Palliative Medicine* 2008;22(2):153-71.
16. Silva AL, Teixeira HJ, Teixeira MJC, et al. The needs of informal caregivers of elderly people living at home: an integrative review. *Scandinavian journal of caring sciences* 2013;27(4):792-803.
17. Clancy RL, Fisher GG, Daigle KL, et al. Eldercare and work among informal caregivers: A multidisciplinary review and recommendations for future research. *Journal of Business and Psychology* 2020;35(1):9-27.
18. Plöthner M, Schmidt K, De Jong L, et al. Needs and preferences of informal caregivers regarding outpatient care for the elderly: a systematic literature review. *BMC geriatrics* 2019;19(1):1-22.
19. Calvó-Perxas L, Vilalta-Franch J, Litwin H, et al. A Longitudinal Study on Public Policy and the health of in-house caregivers in Europe. *Health Policy* 2021

For peer review only - http://bmjopen.bmj.com/site/about/guidelines.xhtml

20. Žalimienė L, Blažienė I, Junevičienė J. What type of familialism is relevant for Lithuania? The case of elderly care. *Journal of Baltic Studies* 2020;51(2):159-78.
21. Kontrimiene A, Sauseriene J, Blazeviciene A, et al. Qualitative research of informal caregivers' personal experiences caring for older adults with mental disorders in Lithuania: A phenomenological approach. 2020
22. Akgun-Citak E, Attepe-Ozden S, Vaskelyte A, et al. Challenges and needs of informal caregivers in elderly care: Qualitative research in four European countries, the TRACE project. *Archives of gerontology and geriatrics* 2020;87:103971.
23. Kontrimiene A, Sauseriene J, Blazeviciene A, et al. Qualitative research of informal caregivers' personal experiences caring for older adults with dementia in Lithuania. *International Journal of Mental Health Systems* 2021;15(1):1-10.
24. Biliunaite I, Dumarkaite A, Kazlauskas E, et al. ICBT program for improving informal caregiver well-being: A qualitative study. *Internet interventions* 2021:100361.
25. Archer J, Reiboldt W, Claver M, et al. Caregiving in Quarantine: Evaluating the Impact of the Covid-19 Pandemic on Adult Child Informal Caregivers of a Parent. *Gerontology and Geriatric Medicine* 2021;7:2333721421990150.
26. Novak M, Guest C. Application of a multidimensional caregiver burden inventory. *The gerontologist* 1989;29(6):798-803.
27. Caserta MS, Lund DA, Wright SD. Exploring the Caregiver Burden Inventory (CBI): further evidence for a multidimensional view of burden. *The International Journal of Aging and Human Development* 1996;43(1):21-34.
28. Greenfield JC, Hasche L, Bell LM, et al. Exploring how workplace and social policies relate to caregivers' financial strain. *Journal of gerontological social work* 2018;61(8):849-66.
29. Lund L, Ross L, Petersen MA, et al. The interaction between informal cancer caregivers and health care professionals: a survey of caregivers' experiences of problems and unmet needs. *Supportive Care in Cancer* 2015;23(6):1719-33.
30. Hengelaar AH, van Hartingsveldt M, Wittenberg Y, et al. Exploring the collaboration between formal and informal care from the professional perspective—A thematic synthesis. *Health & Social Care in the Community* 2018;26(4):474-85.
31. Lévesque L, Ducharme F, Caron C, et al. A partnership approach to service needs assessment with family caregivers of an aging relative living at home: a qualitative analysis of the experiences of caregivers and practitioners. *International Journal of Nursing Studies* 2010;47(7):876-87.
32. Altieri M, Santangelo G. The psychological impact of COVID-19 pandemic and lockdown on caregivers of people with dementia. *The American Journal of Geriatric Psychiatry* 2021;29(1):27-34.
33. Budnick A, Hering C, Eggert S, et al. Informal Caregivers During the COVID-19 Pandemic Perceive Additional Burden: Findings From an Ad-hoc Survey in Germany. 2021
34. Carcavilla N, Pozo AS, González B, et al. Needs of Dementia Family Caregivers in Spain During the COVID-19 Pandemic. *Journal of Alzheimer's Disease* 2021(Preprint):1-5.
35. Mak HW, Bu F, Fancourt D. Mental health and wellbeing amongst people with informal caring responsibilities across different time points during the COVID-19 pandemic: A population-based propensity score matching analysis. *medRxiv* 2021
36. Cohen SA, Kunicki ZJ, Drohan MM, et al. Exploring Changes in Caregiver Burden and Caregiving Intensity due to COVID-19. *Gerontology and Geriatric Medicine* 2021;7:2333721421999279.
37. Biliunaite I, Kazlauskas E, Sanderman R, et al. Internet-Based Cognitive Behavioral Therapy for Informal Caregivers: Randomized Controlled Pilot Trial. *Journal of Medical Internet Research* 2021;23(4):e21466.

For peer review only

For peer review only - http://bmjopen.bmj.com/site/about/guidelines.xhtml

Reporting checklist for cross sectional study.

Based on the STROBE cross sectional guidelines.

Instructions to authors

Complete this checklist by entering the page numbers from your manuscript where readers will find each of the items listed below.

Your article may not currently address all the items on the checklist. Please modify your text to include the missing information. If you are certain that an item does not apply, please write "n/a" and provide a short explanation.

Upload your completed checklist as an extra file when you submit to a journal.

In your methods section, say that you used the STROBE cross sectionalreporting guidelines, and cite them as:

von Elm E, Altman DG, Egger M, Pocock SJ, Gotzsche PC, Vandenbroucke JP. The Strengthening the Reporting of Observational Studies in Epidemiology (STROBE) Statement: guidelines for reporting observational studies.

		Reporting Item	Page Number
**Title and abstract**			
Title	#1a	Indicate the study's design with a commonly used term in the title or the abstract	1

For peer review only

Abstract	#1b	Provide in the abstract an informative and balanced summary of what was done and what was found	2
**Introduction**			
Background / rationale	#2	Explain the scientific background and rationale for the investigation being reported	3-4
Objectives	#3	State specific objectives, including any prespecified hypotheses	4
**Methods**			
Study design	#4	Present key elements of study design early in the paper	4-5
Setting	#5	Describe the setting, locations, and relevant dates, including periods of recruitment, exposure, follow-up, and data collection	4-5
Eligibility criteria	#6a	Give the eligibility criteria, and the sources and methods of selection of participants.	4
	#7	Clearly define all outcomes, exposures, predictors, potential confounders, and effect modifiers. Give diagnostic criteria, if applicable	4-5
Data sources / measurement	#8	For each variable of interest give sources of data and details of methods of assessment (measurement). Describe comparability of	4-5

For peer review only

		assessment methods if there is more than one group. Give information separately for for exposed and unexposed groups if applicable.	
Bias	#9	Describe any efforts to address potential sources of bias	n/a we reflect on selection bias and other potential biases in the limitations section
Study size	#10	Explain how the study size was arrived at	5
Quantitative variables	#11	Explain how quantitative variables were handled in the analyses. If applicable, describe which groupings were chosen, and why	5
Statistical methods	#12a	Describe all statistical methods, including those used to control for confounding	5
Statistical methods	#12b	Describe any methods used to examine subgroups and interactions	n/a
Statistical methods	#12c	Explain how missing data were addressed	n/a – no missing data since completion of the survey was only possible after all questions were answered

For peer review only

Statistical methods	#12d	If applicable, describe analytical methods taking account of sampling strategy	n/a
Statistical methods	#12e	Describe any sensitivity analyses	n/a
**Results**			
Participants	#13a	Report numbers of individuals at each stage of study—eg numbers potentially eligible, examined for eligibility, confirmed eligible, included in the study, completing follow-up, and analysed. Give information separately for for exposed and unexposed groups if applicable.	n/a – study was a cross-sectional survey
Participants	#13b	Give reasons for non-participation at each stage	n/a - study was a cross-sectional survey
Participants	#13c	Consider use of a flow diagram	n/a
Descriptive data	#14a	Give characteristics of study participants (eg demographic, clinical, social) and information on exposures and potential confounders. Give information separately for exposed and unexposed groups if applicable.	5-7
Descriptive data	#14b	Indicate number of participants with missing data for each variable of interest	n/a – no missing data since completion of the survey was only possible after all

For peer review only

			questions were answered
Outcome data	#15	Report numbers of outcome events or summary measures. Give information separately for exposed and unexposed groups if applicable.	5-13
Main results	#16a	Give unadjusted estimates and, if applicable, confounder-adjusted estimates and their precision (eg, 95% confidence interval). Make clear which confounders were adjusted for and why they were included	10-13
Main results	#16b	Report category boundaries when continuous variables were categorized	n/a
Main results	#16c	If relevant, consider translating estimates of relative risk into absolute risk for a meaningful time period	n/a
Other analyses	#17	Report other analyses done—e.g., analyses of subgroups and interactions, and sensitivity analyses	n/a
**Discussion**			
Key results	#18	Summarise key results with reference to study objectives	13-15
Limitations	#19	Discuss limitations of the study, taking into account sources of potential bias or imprecision.	15

For peer review only

		Discuss both direction and magnitude of any potential bias.	
Interpretation	#20	Give a cautious overall interpretation considering objectives, limitations, multiplicity of analyses, results from similar studies, and other relevant evidence.	15
Generalisability	#21	Discuss the generalisability (external validity) of the study results	15
**Other Information**			
Funding	#22	Give the source of funding and the role of the funders for the present study and, if applicable, for the original study on which the present article is based	16

None The STROBE checklist is distributed under the terms of the Creative Commons Attribution License CC-BY. This checklist can be completed online using https://www.goodreports.org/, a tool made by the EQUATOR Network in collaboration with Penelope.ai

For peer review only

BMJ Open

Informal caregiver support needs and burden: a survey in Lithuania

Journal:	*BMJ Open*
Manuscript ID	bmjopen-2021-054607.R1
Article Type:	Original research
Date Submitted by the Author:	15-Nov-2021

Complete List of Authors:	Biliunaite, Ieva; Linköping University, Department of Behavioural Sciences and Learning<br>Kazlauskas, Evaldas; Vilnius University, Institute of Psychology<br>Sanderman, Robbert; University of Groningen, Department of Health Psychology; University of Twente, Department of Psychology, Health and Technology<br>Andersson, Gerhard; Linköping University, Department of Behavioural Sciences and Learning; Linköping University, Department of Biomedical and Clinical Sciences

**Primary Subject Heading**:	Public health
Secondary Subject Heading:	Mental health, Nursing, Palliative care, Health services research
Keywords:	Adult palliative care < PALLIATIVE CARE, PUBLIC HEALTH, COVID-19, International health services < HEALTH SERVICES ADMINISTRATION & MANAGEMENT, Health policy < HEALTH SERVICES ADMINISTRATION & MANAGEMENT, Quality in health care < HEALTH SERVICES ADMINISTRATION & MANAGEMENT

SCHOLARONE™
Manuscripts

I, the Submitting Author has the right to grant and does grant on behalf of all authors of the Work (as defined in the below author licence), an exclusive licence and/or a non-exclusive licence for contributions from authors who are: i) UK Crown employees; ii) where BMJ has agreed a CC-BY licence shall apply, and/or iii) in accordance with the terms applicable for US Federal Government officers or employees acting as part of their official duties; on a worldwide, perpetual, irrevocable, royalty-free basis to BMJ Publishing Group Ltd ("BMJ") its licensees and where the relevant Journal is co-owned by BMJ to the co-owners of the Journal, to publish the Work in this journal and any other BMJ products and to exploit all rights, as set out in our licence.

The Submitting Author accepts and understands that any supply made under these terms is made by BMJ to the Submitting Author unless you are acting as an employee on behalf of your employer or a postgraduate student of an affiliated institution which is paying any applicable article publishing charge ("APC") for Open Access articles. Where the Submitting Author wishes to make the Work available on an Open Access basis (and intends to pay the relevant APC), the terms of reuse of such Open Access shall be governed by a Creative Commons licence – details of these licences and which Creative Commons licence will apply to this Work are set out in our licence referred to above.

Other than as permitted in any relevant BMJ Author's Self Archiving Policies, I confirm this Work has not been accepted for publication elsewhere, is not being considered for publication elsewhere and does not duplicate material already published. I confirm all authors consent to publication of this Work and authorise the granting of this licence.

For peer review only

Informal caregiver support needs and burden: a survey in Lithuania

Ieva Biliunaite[1], MSc; Evaldas Kazlauskas[2], Robbert Sanderman[3,4], Gerhard Andersson[1,5,6]

[1]Department of Behavioural Sciences and Learning, Linköping University, Linköping, Sweden
[2]Center for Psychotraumatology, Institute of Psychology, Vilnius University, Vilnius, Lithuania
[3]Department of Health Psychology, University Medical Center Groningen, University of Groningen, Groningen, Netherlands
[4]Department of Psychology, Health & Technology, University of Twente, Enschede, Netherlands
[5]Department of Biomedical and Clinical Sciences, Linköping University, Linköping, Sweden
[6]Department of Clinical Neuroscience, Karolinska Institutet, Stockholm, Sweden

Corresponding author:
Ieva Biliunaite
Doctoral student
Department of Psychology (PSY)
Department of Behavioural Sciences and Learning,
Linköping University
SE-581 83 Linköping
Sweden
e-mail: ieva.biliunaite@liu.se

For peer review only

ABSTRACT

Introduction: A demand for informal care exists worldwide. Lithuania presents an interesting case example where the need for the informal care is increasing, but relatively little research has been conducted documenting caregivers' experiences and needs.

Objectives: The main objective of this study was to investigate Lithuanian informal caregiver characteristics, support needs and burden. In addition, the impact of the COVID-19 on the caregiver's and care-receiver's well-being was investigated.

Methods: The study was conducted online between May and September 2020. Informal caregivers and individuals with informal caregiving experiences were invited to participate in the survey. The survey questionnaire comprised 38 multiple-choice items including participant demographic characteristics, availability of the support, support needs, well-being, and the impact of the COVID-19 pandemic. In addition, caregiver burden was assessed with the 24-item Caregiver Burden Inventory (CBI).

Results: A total of 226 individuals completed the survey. Most of the participants were women (87.6%). Almost half of the participants (48.7%) were not receiving any support, and a total of 73.9% expressed a need to receive more professional support. Participants were found to experience high burden on the CBI (M=50.21, SD=15.63). Women were found to be significantly more burdened than men (p=.011). Even though many participants experienced psychological problems (55.8%), only 2.2% were receiving any psychological support. Finally, majority of the participants did not experience any changes in their own (63.7%) or the well-being of their care receiver (68.1%) due to the COVID-19 pandemic.

Conclusion: Most of the participants were identified as intensive caregivers experiencing a high burden. A majority did not experience changes in their well-being due to COVID-19. We propose several recommendations for increasing accessibility and availability of support for informal caregivers in Lithuania based on the study findings.

Keywords: adult palliative care; public health; COVID-19; international health services; health policy; quality in health care

Reporting: This report follows the STROBE checklist for cross-sectional studies

Strengths and limitations of this study

- The study was designed to explore needs of growing, yet scarcely researched population of Lithuanian informal caregivers.
- The study targeted a wide range of informal caregivers, providing care in the context of disability, illness, old age, or frailty.
- Taking part in the survey was voluntary and it is likely that the sample is not fully representative.
- Limitations of the self-report data should be considered in interpreting the survey's findings.

For peer review only - http://bmjopen.bmj.com/site/about/guidelines.xhtml

INTRODUCTION

Informal caregivers are individuals, who despite no training or experience, provide care for significant others such as partners, children, siblings, parents, or friends. In Europe, the proportion of individuals involved in some form of informal care ranges between 20% to 44%.[1] Due to the increases in the longevity and demand for long-term care, and the limited resources for formal care, it is likely that more people will need to be involved in informal caregiving in the future.[2] It is evident that informal caregivers are not only important for the management of the long-term care, but that they also carry a substantial economic cost[3] and hence, form a backbone of the health and societal care delivery worldwide.[4]

Informal caregiving experience can vary greatly depending on several factors. For example, the motivation to provide care,[5] intensity of the caregiving,[6] caregivers skills,[7] and the symptoms of the care-receiver are likely to influence the care.[8] It is known that caregiving can lead to positive experiences, such as personal growth or feelings of closeness and intimacy with the care-receiver.[9] At the same time caregivers also experience worse psychological[10] and physical health[11] than non-carers. In addition, they are at risk of loneliness and social isolation,[12] as well as financial difficulties.[13] Accumulation of these negative outcomes can increase caregiver burden, an experience that is described as a combination of the psychological, physical, social, and financial strains.[4] This concerns many women, as they make-up a majority of informal caregivers,[2] and tend to experience worse negative mental health outcomes than male informal caregivers.[4] Altogether, caregiving could be described as a complex experience that often put caregivers at risk of worse psychological health. Providing caregivers with effective support could help to prevent negative outcomes and improve their quality of life, and also improve quality of care for the care-receiver.[14]

Several studies have been conducted over the last years investigating informal caregiver support needs. Some of the more commonly observed needs are the need for information and education in the care provision,[15] a need for better collaboration with health-care professionals,[16] flexible work arrangements,[17] a need for social recognition,[18] as well as the availability of professional support.[16] Despite the commonalties of caregiver needs, research show variation in the caregiver well-being across countries.[19] That is, caregiver needs vary based on the country of residence and the specific cultural and socioeconomic background. For this reason, research on country-specific needs of informal caregivers is needed that consider demographic, cultural, and economical influences.[18]

In this study we focused on Lithuanian informal caregivers. As in many other European countries, in Lithuania demand for the informal care is increasing, while the availability of such care is decreasing. This problem is even more evident in Lithuania, were due to mass emigration and one of the fastest aging populations in Europe, informal care resources are shrinking rapidly.[20] Lithuanian constitution states that it is the duty of the children to take care of their parents.[21] According to the previous findings,[20] more than half of the middle-aged respondents agree with this statement and would prefer to receive informal care themselves. Despite that, social policy measures were previously found to be inadequate in meeting the expectations for the informal care as well as allowing existing informal caregivers to balance their personal, work and caregiving related duties.[20] The available literature regarding Lithuanian informal caregivers' needs is very limited and is mostly based on small scale qualitative findings.[22-23] Some of the needs that were outlined were the need for support regarding care-receiver's as well as own well-being,[23] opportunity for formalized training,[24] flexible working conditions[20] and more professional support and respite services.[25] Although it is evident that Lithuanian informal caregivers experience certain strains, more data is needed for gaining knowledge about their basic characteristics

For peer review only - http://bmjopen.bmj.com/site/about/guidelines.xhtml

and evaluating their challenges so that the following guidelines regarding suitable support options could be proposed. More so, in the light of the COVID-19, as informal caregivers were already identified as a vulnerable group, experiencing more difficulty in providing care and increase in the burden because of the pandemic measures.[26]

Altogether, the main aim of this study was to conduct an online survey investigating Lithuanian informal caregiver characteristics, burden, and general support needs as well as the association between the COVID-19 and the caregiver and care-receiver well-being. More specifically, we aimed at the informal caregivers providing care in the context of disability, illness, old age, or frailty. The results of this survey will be used for warranting healthcare professional, researcher, policy maker and general public's attention towards to the Lithuanian informal caregiver support needs.

METHOD

Design and sample

An online survey was designed to explore characteristics, experiences, and support needs of Lithuanian informal caregivers. To be eligible, participants had to be informal caregivers or have informal caregiving related experience. Also, they needed to be at least 18 years, fluent in Lithuanian language and have internet access via computer, a mobile phone, or a compatible device. We have followed The Strengthening the Reporting of Observational Studies in Epidemiology (STROBE) Statement: guidelines for reporting observational studies.

Participant and Public Involvement

The development of the survey was inspired by the knowledge obtained following the randomized controlled trial as well as follow-up qualitative interviews with the informal caregivers. More specifically, we have identified the need to obtain more knowledge in relation to the caregiver characteristics that could be beneficial for further development and tailoring of the support for the informal caregivers.

Participants and/or the public were not directly involved in the design, or conduct, or reporting or dissemination plans of this research.

Survey development

The survey was conducted in the Lithuanian language. The survey was developed by the authors including a researcher at Vilnius University, with survey items informed by the research questions as well as the current literature.[e.g., 18, 27, 28] More specifically, previous research studies investigating informal caregiver and caregiving related characteristics, well-being, knowledge, and support needs. An established 24-item questionnaire measuring caregiver perceived burden, the Caregiver Burden Inventory (CBI),[29] was also included. The final survey consisted of 62 multiple choice questions. Several items also had an option for free-text answers. A short description of the survey items follows below.

Demographic characteristics and caregiving specifics

There were 14 questions dealing with participant demographic characteristics, such as gender and education. In addition, there were 12 questions in relation to the care-receiver and caregiving in general (e.g., care-recipients age and gender; relation to care-recipient; duration of caregiving).

Caregiving knowledge and support needs

For peer review only - http://bmjopen.bmj.com/site/about/guidelines.xhtml

In this section participants were presented with two questions in relation to their knowledge and five questions in relation to their needs and currently available as well as preferred support options (e.g., *What are the main caregiving related challenges that you experience;* and *Are you receiving caregiving related support?).* Items for this section were inspired by previous research studies investigating informal caregiver use of and need for support.[e.g., 27, 30]

Caregiver burden

In addition, participants were asked to fill in the CBI questionnaire. The CBI consists of five subscales: Physical health, Emotional health, Time Dependency, Development and Social Relationships. Each of the subscales contain five questions with and exception of the Physical Health, which has four questions. Response options range from 0 ('Never') to 4 ('Nearly Always'). A total score is calculated by summing responses (range 0 to 96), with higher total scores indicating higher burden. Sum scores can also be calculated for the subscales separately for Physical Health (range 0 to 16) and for the remaining four subscales (range 0 to 20). Overall, a score of 24 is considered to indicate a need for respite, while scores above 36 - a risk of a burnout.[29] CBI has previously show high reliability.[31] In this sample, reliability coefficients (Cronbach's alpha) were also found to be high: Time Dependency (α= .92), Development (α= .88), Physical Health (α= .87), Emotional Health (α= .84) and Social Relationships (α= .82).

Well-being and support during COVID-19

At the end of the survey, participants were presented with three questions in relation to the COVID-19 pandemic. More specifically, participants were asked how: 1) their own well-being; 2) well-being of the care-recipient and 3) availability of the support have or have not changed due to the pandemic. There were five answer options for answering the third question: *I do not know; improved; did not change; worsened; worsened very much or were not available.* Answer options for the first and second question were presented on a 3-point Likert scale (well-being 1-improved; 2-did not changed; 3-worsened). In answering these questions participants were also requested to indicate type of changes they had observed.

Procedure

The survey was conducted online. Data collection took part between the mid of May and the beginning of September 2020. The link to the survey was disseminated via various social media platforms. The link was also sent to some patient care organizations directly. Interested individuals had to click on the survey link and provide informed consent before taking part in the survey.

Ethics approval for the study was granted by the Vilnius University Psychology Research Ethics Committee, 08-07-2019 No.26. Participation in the survey was voluntary and no monetary compensation was provided.

Statistical Analysis

Data were analysed using IBM SPSS Statistics 25. Descriptive statistics were used for summarizing participant demographic and caregiving related characteristics as well as support needs and the COVID-19 questions responses. Independent samples *t*-tests and One-Way Analysis of Variance (ANOVA) were performed for investigating the association between CBI scores and several demographic characteristics. Multiple linear regression was performed for selected demographic characteristics (as predictors) and CBI total score (as dependent variable). Statistical significance was set at $p<0.05$. When possible, free-text answers were categorized.

For peer review only - http://bmjopen.bmj.com/site/about/guidelines.xhtml

RESULTS

I Informal caregiver demographic characteristics

A total of 226 individuals completed the survey. There were no missing data entry points as the survey could only be submitted when all questions had been answered. Demographic characteristics are presented in the Table 1. As it is evident form the table, majority of the participants were women (87.6%). Given the small proportion of male participants and previously observed gender differences in caregiving prevalence and outcomes, we will report results of both genders separately.

Most of the participants had obtained a university degree (56.2%), were married, or had a partner (69.5%) and were residing in the capital or one of the larger cities in Lithuania (57%). One of the more striking observations was in relation to the occupational status: after starting to provide care a substantial proportion of the participants had either started to be working less than full-time (from 10.6% to 17.3%) or had become unemployed (from 16.8% to 38.9%). This difference was to be statistically significant: $t(225)=-8.69$, $p<0.001$. Regarding self-perceived health, more than half of the sample indicated experiencing physical (58.8%) and psychological health (55.8%) problems.

Table 1. Caregiver characteristics

Participant characteristics	Overall	Women	Men
**Number, n (%)**	226	198 (87.6)	28 (12.4)
**Age caregiver (year): M (SD)**	49.7 (12.7)	49.13 (12.95)	53.89 (10.4)
**Residence: n (%)**			
Capital or one of the larger cities	129 (57)	109 (55.1)	20 (71.4)
Small cities or rural areas	97 (43)	89 (44.9)	8 (28.6)
**Highest education level: n (%)**			
Primary education or vocational training	7 (3.1)	6 (3.0)	1 (3.6)
Secondary education or professional qualification	38 (16.8)	32 (16.2)	6 (21.4)
Applied science or similar	54 (23.9)	50 (25.2)	4 (14.3)
University degree	127 (56.2)	110 (55.6)	17 (60.7)
**Marital status: n (%)**			
Single	32 (14.2)	29 (14.6)	3 (10.7)
Married/partner	157 (69.5)	135 (68.2)	22 (78.6)
Divorced/widowed or other	37 (16.3)	34 (17.2)	3 (10.7)
**Family members: n (%)**			
1-2	81 (35.9)	68 (34.4)	13 (46.5)
3-4	118 (52.2)	106 (53.6)	12 (42.8)
4+	27 (11.9)	24 (12)	3 (10.7)
**Occupational status before caregiving: n (%)**			
Employed full time	164 (72.6)	139 (70.2)	25 (89.3)
Employed part time	24 (10.6)	23 (11.6)	1 (3.6)
Unemployed	38 (16.8)	36 (18.2)	2 (7.1)
**Occupational status after starting caregiving: n (%)**			
Employed full time	99 (43.8)	83 (41.9)	16 (57.1)
Employed part time	39 (17.3)	34 (17.2)	5 (17.9)

For peer review only - http://bmjopen.bmj.com/site/about/guidelines.xhtml

Unemployed	88 (38.9)	81 (40.9)	7 (25)
**Financial situation: n (%)**			
Cannot afford enough food	42 (18.6)	40 (20.2)	2 (7.1)
Enough for food, but not for bigger purchases (e.g.: TV)	83 (36.7)	70 (35.4)	13 (46.4)
Enough for bigger, but not very big purchases (e.g.: a flat)	92 (40.7)	81 (40.9)	11 (39.4)
Everything is affordable	9 (4.0)	7 (3.5)	2 (7.1)
**Health problems caregiver: n (yes %)**			
Physical health problems	133 (58.8)	118 (59.6)	15 (53.6)
Psychological health problems	126 (55.8)	115 (58.1)	11 (39.3)
**Self-rated well-being over last four weeks: n (%)**			
Either very good or good	42 (18.6)	35 (17.6)	7 (25)
Neither good nor bad	82 (36.3)	70 (35.4)	12 (42.9)
Not very good or bad	102 (45.1)	93 (47)	9 (32.1)

II Caregiving specifics

Care-receiver's characteristics as well as caregiving intensity-related information are presented in Table 2. Most of the care-receivers were women (68.1%), and the mean age was 76 years (SD=19.85). The care-receiver's age varied, with the youngest being 5 years and oldest 99 years. However, only 5.8% of informal caregivers were providing care for 0-18 years old, and a majority (54%) provided care in the age range of 80–100 years old. Recipients were most commonly providing care for their parent (father or mother) (57.1%). Among the types of relations as categorized from the free-text answers and not mentioned in the table, the most common for the recipients was to be a grandmother (5%). Most of the care-receivers had dementia (22.6%), a previous experience of stroke or myocardial infarction (15%) or needed assistance because of old age (13.7%). Regarding reasons for care provision, two of the most frequent reasons were own initiative (23%) and having no other family member available for care provision (23%). Other common reasons stated in the free-text boxes were the care-receiver requesting care (6.2%) and being the parent of the care-receiver (6.2%).

Almost half of the participants had provided care for more than four years (48.2%), five to seven days per week (79.6%), and either three to seven (32.7%) or more than 12 hours per day (35.4%). In addition, 8% of the participants used free-text answer option to indicate that the care-receiver was fully dependent on their support.

Table 2. Caregiving information

Caregiving related information	Overall	Women	Men
**Gender care-recipient: n (%)**		154 (68.1)	72 (31.9%)
**Age care-recipient (year): n (%)**	71.38 (23.31)	76.27 (19.85)	60.93 (26.65)
**Relation to care-recipient: n (%)**			
Husband/wife/partner	23 (10.2)	13 (6.6)	10 (35.7)
Father/mother	129 (57.1)	116 (58.6)	13 (46.4)
Parent-in-law/uncle/auntie	23 (10.2)	19 (9.6)	4 (14.3)
Brother/sister	5 (2.2)	5 (2.5)	0
Daughter/son	25 (11.1)	25 (12.6)	0
Other	21 (9.3)	20 (10.1)	1 (3.6)

For peer review only

For peer review only - http://bmjopen.bmj.com/site/about/guidelines.xhtml

**Main reason for caregiving: n (%)**			
Old age	31 (13.7)	27 (13.6)	4 (14.3)
Dementia	51 (22.6)	45 (22.7)	6 (21.5)
Stroke/ Myocardial infarction	34 (15)	32 (16.3)	2 (7.1)
Amyotrophic lateral sclerosis (ALS)	12 (5.3)	6 (3)	6 (21.5)
Cerebral palsy	11 (4.9)	11 (5.6)	0
Cancer	10 (4.4)	8 (4)	2 (7.1)
Other	77 (34.1)	69 (34.8)	8 (28.5)
**Caregiver resides with care receiver: n (yes %)**	163 (72.1)	142 (71.7)	21 (75)
**Caregiving circumstances: n (single caregiver %)**	92 (40.7)	76 (38.4)	16 (57.1)
**Reasons for providing care: n (%)**			
Own initiative	52 (23)	44 (22.2)	8 (28.6)
Due to close living proximity to the care-receiver	26 (11.5)	21 (10.6)	5 (17.9)
There were no other family members available for caregiving	52 (23)	46 (23.2)	6 (21.4)
This what was agreed on together with other family members	41 (18.1)	39 (19.7)	2 (7.1)
Other	55 (24.4)	48 (24.3)	7 (25)
**Time caring: n (in months %)**			
<12	47 (20.8)	45 (22.7)	2 (7.1)
12-24	36 (16)	28 (14.1)	8 (28.6)
24-48	34 (15)	32 (16.2)	2 (7.1)
48+	109 (48.2)	93 (47)	16 (57.1)
**Time per week: n (in days %)**			
1-2	26 (11.5)	23 (11.6)	3 (10.7)
3-4	20 (8.8)	17 (8.6)	3 (10.7)
5-7	180 (79.6)	158 (79.8)	22 (78.6)
**Time per day: n (in hours %)**			
3<	51 (22.6)	40 (20.2)	11 (39.3)
3-7	74 (32.7)	64 (32.3)	10 (35.7)
8-11	21 (9.3)	20 (10.1)	1 (3.6)
12+	80 (35.4)	74 (37.4)	6 (21.4)
**Helping care-receiver with basic activities of daily living (ADLs)[a]: n (yes %)**			
Bathing	175 (77.4)	160 (80.8)	15 (53.6)
Brushing teeth	102 (45.1)	95 (48)	7 (25)
Dressing	156 (69)	140 (70.7)	16 (57.1)
Eating	160 (70.8)	143 (72.2)	17 (60.7)
Moving	155 (68.6)	134 (67.7)	21 (75)
Toilet needs	130 (57.5)	116 (58.6)	14 (50)
Maintaining general hygiene (e.g., cutting nails)	189 (83.6)	170 (85.9)	19 (67.9)
**Helping care-receiver with instrumental activities of daily living (IADLs)[a]: n (yes %)**			
Using telephone	100 (44.2)	87 (43.9)	13 (46.4)
Laundry	167 (73.9)	143 (72.2)	24 (85.7)
Shopping	170 (75.2)	146 (73.7)	24 (85.7)

For peer review only - http://bmjopen.bmj.com/site/about/guidelines.xhtml

Transportation	158 (69.9)	134 (67.7)	24 (85.7)
Cooking	172 (76.1)	148 (74.7)	24 (85.7)
Medication	164 (72.6)	147 (74.2)	17 (60.7
Household	179 (79.2)	152 (76.8)	27 (96.4)
Financial management	139 (61.5)	122 (61.6)	17 (60.7)

[a]Possible to choose more than one response option.

III Informal caregiver knowledge and support needs

Several aspects in relation to the informal caregivers knowledge and needs were identified (Table 3). Almost half of the participants (47.3%) reported no specific knowledge about the disorder of the care-recipient, and more than half (55.3%) reported no knowledge about how to provide care in general. Consequently, a majority wished to receive more professional support in their role as caregivers (73.9%). Less time for oneself and changes in physical and mental health were identified as the two most prominent challenges, 84.5% and 76.5% respectively. Regarding support, most of participants either did (34.5%) or did not look for profession support (35%). In turn, almost half of the participants reported that they were not receiving any caregiving-related support (48.7%). Regarding the ones receiving support, financial aid was the most mentioned (33.6%). Only 2.2% of the participants received psychological help. Participants reported that their situation would improve if caregiving was recognised as part of working experience (55.3%), if they would receive financial (46.5%) or professional support (45.1%), and more days for respite (44.2%).

Table 3. Caregiver's knowledge and support needs

Participant characteristics	Overall	Women	Men
**Number, n (%)**	226	198 (87.6)	28 (12.4)
**Prior care provision knowledge regarding the disorder(s) of care-receiver: n (no knowledge %)**	107 (47.3)	96 (48.5)	11 (39.3)
**Prior care provision knowledge in general: n (no knowledge %)**	125 (55.3)	108 (54.5)	17 (60.7)
**Would you like to receive more professional support with caregiving (medical, social etc): n (yes %)**	167 (73.9)	143 (72.2)	24 (85.7)
**Personal difficulties experienced by caregivers[a]: n (%)**			
Less time for one-self	191 (84.5)	169 (85.4)	22 (78.6)
Changes in sleep quality	128 (56.6)	114 (57.6)	14 (50)
Changes in relationships with other people	122 (54)	110 (55.6)	12 (42.9)
Changes in financial situation	107 (47.3)	95 (48)	12 (42.9)
Changes in physical or psychological health	173 (76.5)	156 (78.8)	17 (60.7)
**Have you searched for caregiving related support[a]: n (%)**			
Have not searched	79 (35)	67 (33.8)	12 (42.9)
Yes, searched financial support	54 (23.9)	45 (22.7)	9 (32.1)
Yes, searched for own well-being support	64 (28.3)	59 (29.8)	5 (17.9)

For peer review only - http://bmjopen.bmj.com/site/about/guidelines.xhtml

Yes, looked for professional support for conducting caregiving tasks	78 (34.5)	69 (34.8)	9 (32.1)
**Receiving caregiving related support[a]: n (%)**			
Not receiving support	110 (48.7)	98 (49.5)	12 (42.9)
Receiving financial support	76 (33.6)	68 (34.3)	8 (28.6)
Receiving psychological support	5 (2.2)	5 (2.5)	0
Receiving professional support for caregiving tasks	27 (11.9)	22(11.1)	5 (17.9)
**My situation would improve if[a]: n (%)**			
I would receive psychological support	73 (32.3)	67 (33.8)	6 (21.4)
I would receive professional caregiving related support	102 (45.1)	88 (44.4)	14 (50)
I would receive more respite days	100 (44.2)	90 (45.5)	10 (35.7)
I would receive financial support	105 (46.5)	87 (43.9)	18 (64.3)
I would receive more information about the care-provision and specific disorder	59 (26.1)	53 (26.8)	10 (35.7)
I would receive more support from people in my close environment	59(26.1)	56 (28.3)	3 (10.7)
If time spent caregiving would add to the years of working	125 (55.3)	109 (55.1)	16 (57.1)
Other			

[a]Possible to choose more than one response option.

IV Informal caregiver burden

Mean scores, standard deviations, and gender differences regarding scores on the CBI and the separate sub-scales are presented in Table 4. Since all the subscales have five items each except for the Physical Health subscale, which has four, the scores for this subscale were multiplied by 1.25.[29] Overall, participants displayed high mean score on the CBI (M=50.21, SD=15.63), with highest mean score on the subscale Time Dependency (M=16.15, SD=4.21). As illustrated in Table 4, women scored significantly higher on the overall CBI score (*p*=.011) as well as on the subscales Development (*p*=.035) and Physical Health (*p*=.002).

Table 4. Means, standard deviations and independent samples *t*-test results for CBI total score and separate subscales.

Scale	Mean (SD)			*t*	*p*
	Overall	Women	Men		
CBI[a] total score	50.21 (15.63)	51.2 (15.41)	43.18 (15.68)	-2.57	.011
Time Dependency	16.15 (4.21)	16.30 (4.16)	15.07 (4.48)	-1.45	.149
Development	12.77 (4.85)	13.03 (4.86)	10.96 (4.44)	-2.12	.035
Physical Health	11.07(4.85)	11.44 (4.76)	8.44 (4.75)	-3.12	.002

For peer review only - http://bmjopen.bmj.com/site/about/guidelines.xhtml

Emotional Health	4.93 (4.04)	5.05 (4.04)	4.11 (4.0)	-1.15	.251
Social relationships	7.5 (4.71)	7.68 (4.58)	6.29 (5.45)	-1.47	.144

[a]CBI - Caregiver Burden Inventory.

Independent samples *t*-tests or ANOVAs were performed when analysing demographic as well as caregiving-related characteristics (with exception of the care-receiver symptoms, which was not included due to the many categories) in relation to CBI total scores (Table 5). Eight variables (nine if gender is included) were found to be associated with increased CBI scores: physical (p<.001) or psychological (p<.001) health complaints, poorer self-rated well-being (p<.001), residing with the care-receiver (p<.001) and caring for longer and with higher intensity (p<.001). Also, informal caregivers who started providing care as there were no other family members to help were found to be significantly more burdened than individuals who took up this task following own initiative (p=.001).

Table 5. Caregiver burden associations with sociodemographic and informal caregiver study variables

Variable	CBI[a], M (SD)	*t* or *F*[b]	*p*
**Age caregiver**			
18-39	47.07 (2.15)	1.61	.203
40-59	51.66 (15.86)		
60-80	48.98 (16.09)		
**Residence**			
Capital or one of the larger cities	48.9 (17.31)	-1.51	.131
Small cities or rural areas	51.95 (12.96)		
**Education**			
Primary education or vocational training	49.71 (15.91)	0.78	.504
Secondary education or professional qualification	48.76 (12.52)		
Applied science or similar	53.02 (14.89)		
University degree	49.47 (16.75)		
**Marital status**			
Single	49.09 (12.57)	0.15	.862
Married/partner	50.21 (15.56)		
Divorced/widowed or other	51.16 (18.44)		
**Family members**			
1-2	49.09 (15.37)	1.93	.148
3-4	51.94 (15.75)		
4+	46.0 (15.33)		
**Financial situation**			
Cannot afford enough food	52.67 (18.82)	0.42	.737
Enough for food, but not for bigger purchases (e.g.: TV)	49.7 (13.60)		
Enough for bigger, but not very big purchases (e.g.: a flat)	49.61 (15.79)		
Everything is affordable	49.56 (16.95)		
**Current occupational status**			
Employed full time	48.52 (16.07)	1.05	.352
Employed part time	51.90 (17.84)		
Unemployed	51.36 (14.01)		
**Physical health problems caregiver**			

For peer review only - http://bmjopen.bmj.com/site/about/guidelines.xhtml
For peer review only

No	45.82 (15.74)	-3.59	<.001
Yes	53.28 (14.86)		
**Psychological health problems caregiver**			
No	43.8 (14.83)	-5.86	<.001
Yes	55.29 (14.38)		
**Self-rated well-being over last four weeks**			
Either very good or good	36.88 (15.18)	22.88	<.001
Neither good nor bad	52.09 (12.36)		
Not very good or bad	54.19 (15.35)		
**Gender care-receiver**			
Men	49.04 (15.53)	-0.77	.444
Women	50.75 (16.14)		
**Age care-receiver**			
0-18	53.31 (5.22)	1.14	.337
19-39	54.59 (14.62)		
40-59	44.45 (8.89)		
60-79	50.19 (17.43)		
80-100	50.22 (16.39)		
**Relation care-receiver**			
Husband/wife/partner	45.39 (13.71)	1.72	.132
Father/mother	51.19 (15.87)		
Parent-in-law/uncle/auntie	46.87 (20.09)		
Brother/sister	49.0 (10.56)		
Daughter/son	56.04 (8.24)		
Other	46.48 (16.70)		
**Individual is the only caregiver**			
Yes	52.37 (14.30)	1.73	.085
No	48.72 (16.37)		
**Reason why one started providing care**			
Own initiative	44.17 (16.50)	4.59	.001
Due to the proximity to the care-receiver	47.31 (19.70)		
No other family members available	56.46 (13.17)		
Decided with family members	50.27 (15.32)		
Other	51.33 (12.88)		
**Receiving of caregiving related support**			
Receiving support	48.89 (16.99)	1.31	.191
Not receiving support	51.6 (14.0)		
**Residing with the care-receiver**			
Yes	52.9 (13.28)	8.42	<.001
No	43.24 (18.91)		
**Time Caring: months**			
<12	45.17 (19.20)	7.99	<.001
12-24	42.56 (14.72)		
24-48	51.56 (15.44)		
48+	54.49 (12.62)		
**Time week: days**			
1-2	39.04 (19.87)	8.12	<.001
3-4	49.90 (18.14)		
5-7	51.86 (14.01)		
**Time day: hours**			
<3	39.90 (19.07)	11.82	<.001
3-7	52.12 (13.87)		

For peer review only

For peer review only - http://bmjopen.bmj.com/site/about/guidelines.xhtml

8-11	50.0 (9.85)
12+	55.06 (13.04)

[a]CBI - Caregiver Burden Inventory.
[b]Either Independent samples t-tests or One-Way Analysis of Variance (ANOVA) were performed dependently on the number of categories.

We ran a hierarchical multiple linear regression including the significant predictors presented in Table 5. Out of the nine entered predictors, four made a significant independent contribution to CBI total score: self-rated well-being (*p*=.001), caregiving duration in months (*p*=.006), caregiver's gender (*p*=.046), and experience of the psychological health problems (p=.001) (Table 6, Block 1). We ran the regression again with all four variables included. All variables were found to contribute to the model significantly, explaining 27.3% of variance in the CBI scores (Table 6, Block 2).

Table 6. Multiple linear regression results with demographic characteristics as predictors and CBI as an outcome variable

	Block 1				
**Included variables**	**B [95% CI]**	**SE B**	**β**	**p**	**R^2**
Self-rated well-being over last four weeks	4.05 [1.77, 6.32]	1.15	0.23	.001	0.324
Time day: hours	0.94 [-0.78, 2.65]	0.87	0.07	.283	
Time week: days	2.63 [-0.62, 5.89]	1.65	0.11	.112	
Time Caring: months	2.17 [0.64, 3.70]	0.78	0.17	.006	
Reason why one started providing care	0.87 [-0.32, 2.06]	0.60	0.08	.151	
Residing with the care-receiver	2.46 [-2.62, 7.54]	2.58	0.07	.341	
Gender caregiver	5.50 [0.09 10.90]	2.74	0.12	.046	
Physical health problems caregiver	1.43 [-2.46, 5.32]	1.97	0.05	.469	
Psychological health problems caregiver	6.33 [2.48, 10.18]	1.95	0.20	.001	
	**Block 2**				
**Included variables**	**B [95% CI]**	**SE B**	**β**	**p**	**R^2**
Self-rated well-being over last four weeks	4.79 [2.64, 6.94]	1.09	0.27	<.001	0.273
Time Caring: months	3.02 [1.54, 4.50]	0.75	0.23	<.001	
Gender caregiver	6.26 [0.83, 11.69]	2.76	0.13	.024	
Psychological health problems caregiver	6.77 [2.90, 10.63]	1.96	0.22	.001	

V Wellbeing during Covid-19

Out of the 226 participants, a majority indicated that neither their own (63.7%) nor the care-receiver's well-being (68.1%) had changed during the COVID-19 pandemic. Regarding the

For peer review only - http://bmjopen.bmj.com/site/about/guidelines.xhtml

availability of the care-related support, 27.4% indicated a decrease and 32.3% a very big decrease in the availability of support.

DISCUSSION

The aim of this study was to investigate Lithuanian informal caregiver characteristics, support needs, burden, and the impact of the COVID-19 on the well-being. Overall, informal caregivers in this survey displayed high burden, high involvement in the care-provision and limited access to the support options. Most of the participants indicated no changes in their well-being due to the COVID-19. We further discuss the findings as well as the limitations of this study below.

Caregiver and caregiving related characteristics

The mean age of the participants in the survey (M=49) could be deemed compatible with median age of Lithuanian citizens (M=45)[32] and is in line with the research literature indicating that most of the informal care in Lithuania, as in other parts of the world, is carried out by women in their fifties.[6] More than half of the participants reported either psychological or physical health problems suggesting that caregivers are at risk of poor well-being.[10, 11, 19] We also observed a significant decrease in the proportion of caregivers working full-time after the start of the care provision. Current labour market-related measures for informal caregivers in Lithuania could be described as limited and insufficient.[20] This might explain why participants in our study had to move from working full-time to either not working at all or working reduced number of hours. Having to reduce work hours due to the caregiving duties alone has previously been found to have a negative effect on the caregiver's psychological well-being.[33] Further efforts to improve current labour market-related measures are most likely be needed to prevent such risks.

Individuals who provide care for 11 or more hours per week have previously been defined as intensive caregivers.[6] In our sample, 77.4% of all the participants fell into this category. Most of these caregivers provided care for five to seven days per week, and at the time of the survey completion, for four or more years. Mental health consequences have previously been found to be even more severe for intensive caregivers,[6] a finding that could at least partly explain the sample's high overall scores on the CBI measure. In line with this, most of the participants indicated that they would like to receive more professional support. In terms of available support, current day care and nursing home services as well as respite services for the informal caregivers in Lithuania could be described as very limited.[20] This suggests that further policy measures for improving both, availability, and accessibility of such services are needed.

Caregiver knowledge and support needs

Most of the participants started providing care without having any general or receiver symptom- specific knowledge about caregiving. Informal caregivers in Lithuania often have to learn about the care provision through own experience.[24] As a consequence, they often experience feelings of anxiety and uncertainty. In addition, almost half of the participants did not receive any support in their caregiving. Among those who had support, a majority received financial support. Time spent for caregiving being counted as work experience was the most favoured suggestion by the caregivers. In addition, a majority indicated that financial and professional support would improve their situation. Interestingly, approximately one third of the participants indicated that they had not searched for support. One explanation could be that they did not know which support is available or how to access it.[24] Prior negative experiences of interactions with health care professionals could also influence health care seeking. A recent qualitative study on Lithuanian informal caregivers

For peer review only - http://bmjopen.bmj.com/site/about/guidelines.xhtml

reported that some caregivers experienced difficulty in communicating with the healthcare professionals.[22] Studies in other countries have also found that carers experience dissatisfaction with the health care providers in terms of information provision, treatment optimization, involvement of the caregiver and management of caregivers' own health.[34] As a solution, additional training could be offered to the professionals about guiding and supporting informal caregivers.[35] Early initiation of the contact with the caregivers could also be useful. This might be especially important for cases in which help-seeking behaviour conflicts with caregivers' values[36] or caregivers express high needs for continuous or frequent support.

Caregiver burden

In line with the previous literature,[4, 6] we found that women participants experienced a higher burden than the men. Participants overall scored the highest on the Time Dependency subscale of the CBI which mirrors a large time investment on caregiving duties. This was further outlined by the regression analyses, in which being a woman, longer caregiving duration, poorer self-rated well-being and psychological health problems were significant predictors of higher CBI total scores. The question of what type of psychological support options informal caregivers would prefer remains. As identified in the recent qualitative Lithuanian informal caregiver study[24] access to peer support groups as well as internet-based intervention programs could have potential in reducing caregiver psychological burden. Further research into these matters is encouraged.

COVID-19

Contrary to our expectations and recent researcher findings[26, 37-39] most of the informal caregivers did not report any changes in own or care-receivers well-being because of the COVID-19 pandemic. One possible explanation could stem from the finding that in comparison to other European countries, in Lithuania, comparably lower number of cases as well as COVID-19 related deaths were reported during the first wave of the pandemic.[40] Lithuanian government has also taken early preventative measures which were deemed as innovative and promising in bettering the social policies.[41] Alternatively, higher appreciation of the life at the start of the pandemic could be another explanation why no changes were observed.[42] As outlined recently, changes in the caregiver burden during the pandemic might be rather complex and vary by gender.[43] Therefore, it is possible that our questions did not capture the complexity of such changes. Future studies are needed to evaluate the impact of the pandemic on informal caregivers.

Study limitations

There are limitations to be addressed. Firstly, our sample might not be representative for Lithuanian caregivers as participants are likely to have higher computer literacy and motivation to participate in online research. Even though internet access is widely spread throughout the country, people in their fifties were found to access the internet less often than the younger age groups.[44] In addition, submission of the survey responses was only possible upon completion of all items. This could have had an influence on participant motivation to complete the survey and hence, add to the sample selection bias. Secondly, caregiver knowledge and support needs as well as changes in well-being during the COVID-19 were investigated by the use of only few items. Therefore, findings should be replicated using established and validated questionnaires. Thirdly, our study was cross-sectional and did only investigate caregiver needs at a certain point in time. Longitudinal data should be collected for more comprehensive evaluation of the possible fluctuations in well-being and support needs over time. Also, for the COVID-19, considering the changing nature of the

For peer review only - http://bmjopen.bmj.com/site/about/guidelines.xhtml

pandemic. Finally, it must be emphasized that due to the cross-sectional design of this research study, all findings are descriptive, indicating that no causal inferences should be drawn.

Conclusion and recommendations

Based on the results of our sample we conclude that the Lithuanian informal caregivers, in relation to caregivers in other European countries, experience high burden and unmet practical as well as psychological support needs. We outline here a few points that could be focused on by policy makers, healthcare professionals and researchers. Firstly, current labour market policies are insufficient in allowing caregivers to balance caregiving, work, and personal life. To prevent possible negative financial and psychological health consequences for the caregivers, further emphasis should be put on adapting current policies. Secondly, we found the caregivers to express the need for information and practical support. More accessible information sources and better guidance from health professionals could be offered. Lastly, participants in our study were found to experience high caregiver burden. Due to the low coverage and accessibility of psychological support options, we encourage researchers to develop innovative support measures, such as online support groups or psychological support interventions.[45]

We conclude that supporting informal caregivers is crucial not only for the individual, but also on a societal level. Meeting these needs is important from the start and throughout the caregiving experience.

Acknowledgments

We thank George Vlaescu and Austeja Dumarkaite for the help with the study. We also thank all the individuals and organizations that willingly helped with the dissemination of the survey.

Contributors

All authors contributed to the conception and study design of the study. IB and EK contributed to the data collection. IB and GE analysed and interpreted the data. IB drafted the manuscript. EK, RS and GA critically revised the paper. All authors approved the final version of the manuscript.

Funding

This project has received funding from the European Union’s Horizon 2020 research and innovation programme under the Marie Skłodowska-Curie grant agreement No 814072 and is part of The European Training Network on Informal Care (ENTWINE).

Competing interests

None declared.

Data availability statement

Anonymized data will be stored at Linköping university for 10 years. It will be available upon reasonable request in excel format after the publication of the manuscript. Primary investigator (Prof Gerhard Andersson, Linköping University; gerhard.andersson@liu.se) should be contacted for requesting about the data.

Ethics approval statement

For peer review only - http://bmjopen.bmj.com/site/about/guidelines.xhtml

Ethics approval for the study was granted by the Vilnius University Psychology Research Ethics Committee, 08-07-2019 No.26.

Abbreviations

CBI: Caregiver Burden Inventory

For peer review only

For peer review only - http://bmjopen.bmj.com/site/about/guidelines.xhtml

References

1. Verbakel E. How to understand informal caregiving patterns in Europe? The role of formal long-term care provisions and family care norms. *Scandinavian journal of public health* 2018;46(4):436-47.
2. Talley RC, Crews JE. Framing the public health of caregiving. *American Journal of Public Health* 2007;97(2):224-28.
3. Rabarison KM, Bouldin ED, Bish CL, et al. The economic value of informal caregiving for persons with dementia: Results from 38 states, the District of Columbia, and Puerto Rico, 2015 and 2016 BRFSS. *American journal of public health* 2018;108(10):1370-77.
4. Revenson T, Griva K, Luszczynska A, et al. Caregiving in the illness context: Springer 2016.
5. Quinn C, Clare L, Woods RT. The impact of motivations and meanings on the wellbeing of caregivers of people with dementia: a systematic review. *International Psychogeriatrics* 2010;22(1):43.
6. Verbakel E, Tamlagsrønning S, Winstone L, et al. Informal care in Europe: findings from the European Social Survey (2014) special module on the social determinants of health. *The European Journal of Public Health* 2017;27(suppl_1):90-95.
7. Mollica MA, Litzelman K, Rowland JH, et al. The role of medical/nursing skills training in caregiver confidence and burden: a CanCORS study. *Cancer* 2017;123(22):4481-87.
8. Harding R, Gao W, Jackson D, et al. Comparative analysis of informal caregiver burden in advanced cancer, dementia, and acquired brain injury. *Journal of pain and symptom management* 2015;50(4):445-52.
9. Cross AJ, Garip G, Sheffield D. The psychosocial impact of caregiving in dementia and quality of life: a systematic review and meta-synthesis of qualitative research. *Psychology & health* 2018;33(11):1321-42.
10. Sallim AB, Sayampanathan AA, Cuttilan A, et al. Prevalence of mental health disorders among caregivers of patients with Alzheimer disease. *Journal of the American Medical Directors Association* 2015;16(12):1034-41.
11. Berglund E, Lytsy P, Westerling R. Health and wellbeing in informal caregivers and non-caregivers: a comparative cross-sectional study of the Swedish general population. *Health and Quality of Life Outcomes* 2015;13(1):1-11.
12. Vasileiou K, Barnett J, Barreto M, et al. Experiences of loneliness associated with being an informal caregiver: a qualitative investigation. *Frontiers in Psychology* 2017;8:585.
13. Cipollone A, Patacchini E, Vallanti G. Female labour market participation in Europe: novel evidence on trends and shaping factors. *IZA Journal of European Labor Studies* 2014;3(1):1-40.
14. Litzelman K, Kent EE, Mollica M, et al. How does caregiver well-being relate to perceived quality of care in patients with cancer? Exploring associations and pathways. *Journal of Clinical Oncology* 2016;34(29):3554.
15. Docherty A, Owens A, Asadi-Lari M, et al. Knowledge and information needs of informal caregivers in palliative care: a qualitative systematic review. *Palliative Medicine* 2008;22(2):153-71.
16. Silva AL, Teixeira HJ, Teixeira MJC, et al. The needs of informal caregivers of elderly people living at home: an integrative review. *Scandinavian journal of caring sciences* 2013;27(4):792-803.
17. Clancy RL, Fisher GG, Daigle KL, et al. Eldercare and work among informal caregivers: A multidisciplinary review and recommendations for future research. *Journal of Business and Psychology* 2020;35(1):9-27.
18. Plöthner M, Schmidt K, De Jong L, et al. Needs and preferences of informal caregivers regarding outpatient care for the elderly: a systematic literature review. *BMC geriatrics* 2019;19(1):1-22.
19. Calvó-Perxas L, Vilalta-Franch J, Litwin H, et al. A Longitudinal Study on Public Policy and the health of in-house caregivers in Europe. *Health Policy* 2021

For peer review only - http://bmjopen.bmj.com/site/about/guidelines.xhtml

20. Žalimienė L, Blažienė I, Junevičienė J. What type of familialism is relevant for Lithuania? The case of elderly care. *Journal of Baltic Studies* 2020;51(2):159-78.
21. Republic of Lithuania, “Constitution of the Republic of Lithuania.” Adopted by citizens of the Republic of Lithuania in the Referendum of 25 October 1992.

22. Kontrimiene A, Sauseriene J, Blazeviciene A, et al. Qualitative research of informal caregivers’ personal experiences caring for older adults with mental disorders in Lithuania: A phenomenological approach. 2020
23. Akgun-Citak E, Attepe-Ozden S, Vaskelyte A, et al. Challenges and needs of informal caregivers in elderly care: Qualitative research in four European countries, the TRACE project. *Archives of gerontology and geriatrics* 2020;87:103971.
24. Kontrimiene A, Sauseriene J, Blazeviciene A, et al. Qualitative research of informal caregivers’ personal experiences caring for older adults with dementia in Lithuania. *International Journal of Mental Health Systems* 2021;15(1):1-10.
25. Biliunaite I, Dumarkaite A, Kazlauskas E, et al. ICBT program for improving informal caregiver well-being: A qualitative study. *Internet interventions* 2021:100361.
26. Archer J, Reiboldt W, Claver M, et al. Caregiving in Quarantine: Evaluating the Impact of the Covid-19 Pandemic on Adult Child Informal Caregivers of a Parent. *Gerontology and Geriatric Medicine* 2021;7:2333721421990150.
27. Carroll L, Chippior J, Karmali S, et al. We Are Caregivers: Social Identity Is Associated with Lower Perceived Stress among Rural Informal Caregivers. *Canadian Journal on Aging/La Revue canadienne du vieillissement* 2019;38(1):59-75.
28. Dixe MdACR, da Conceição Teixeira LF, Areosa TJTCC, et al. Needs and skills of informal caregivers to care for a dependent person: a cross-sectional study. *BMC geriatrics* 2019;19(1):1-9.

29. Novak M, Guest C. Application of a multidimensional caregiver burden inventory. *The gerontologist* 1989;29(6):798-803.
30. Peeters JM, Van Beek AP, Meerveld JH, et al. Informal caregivers of persons with dementia, their use of and needs for specific professional support: a survey of the National Dementia Programme. *BMC nursing* 2010;9(1):1-8.
31. Caserta MS, Lund DA, Wright SD. Exploring the Caregiver Burden Inventory (CBI): further evidence for a multidimensional view of burden. *The International Journal of Aging and Human Development* 1996;43(1):21-34.
32. Worldometer, Lithuanian population (live). Available: https://www.worldometers.info/world-population/lithuania-population/ [Accessed 9 November 2021]
33. Greenfield JC, Hasche L, Bell LM, et al. Exploring how workplace and social policies relate to caregivers’ financial strain. *Journal of gerontological social work* 2018;61(8):849-66.
34. Lund L, Ross L, Petersen MA, et al. The interaction between informal cancer caregivers and health care professionals: a survey of caregivers’ experiences of problems and unmet needs. *Supportive Care in Cancer* 2015;23(6):1719-33.
35. Hengelaar AH, van Hartingsveldt M, Wittenberg Y, et al. Exploring the collaboration between formal and informal care from the professional perspective—A thematic synthesis. *Health & Social Care in the Community* 2018;26(4):474-85.
36. Lévesque L, Ducharme F, Caron C, et al. A partnership approach to service needs assessment with family caregivers of an aging relative living at home: a qualitative analysis of the experiences of caregivers and practitioners. *International Journal of Nursing Studies* 2010;47(7):876-87.
37. Altieri M, Santangelo G. The psychological impact of COVID-19 pandemic and lockdown on caregivers of people with dementia. *The American Journal of Geriatric Psychiatry* 2021;29(1):27-34.

For peer review only - http://bmjopen.bmj.com/site/about/guidelines.xhtml

38. Budnick A, Hering C, Eggert S, et al. Informal Caregivers During the COVID-19 Pandemic Perceive Additional Burden: Findings From an Ad-hoc Survey in Germany. 2021
39. Carcavilla N, Pozo AS, González B, et al. Needs of Dementia Family Caregivers in Spain During the COVID-19 Pandemic. *Journal of Alzheimer's Disease* 2021(Preprint):1-5.
40. Sannigrahi S, Pilla F, Basu B, et al. Examining the association between socio-demographic composition and COVID-19 fatalities in the European region using spatial regression approach. *Sustainable cities and society* 2020;62:102418.
41. Aidukaite J, Saxonberg S, Szelewa D, et al. Social policy in the face of a global pandemic: Policy responses to the COVID-19 crisis in Central and Eastern Europe. *Social Policy & Administration* 2021;55(2):358-73.
42. Mak HW, Bu F, Fancourt D. Mental health and wellbeing amongst people with informal caring responsibilities across different time points during the COVID-19 pandemic: A population-based propensity score matching analysis. *medRxiv* 2021
43. Cohen SA, Kunicki ZJ, Drohan MM, et al. Exploring Changes in Caregiver Burden and Caregiving Intensity due to COVID-19. *Gerontology and Geriatric Medicine* 2021;7:2333721421999279.
44. Liubinienė V, Thunqvist DP. Media literacy and digital divide: A cross-cultural case study of Sweden and Lithuania. *Creativity Studies* 2015;8(2):134-48.
45. Biliunaite I, Kazlauskas E, Sanderman R, et al. Internet-Based Cognitive Behavioral Therapy for Informal Caregivers: Randomized Controlled Pilot Trial. *Journal of Medical Internet Research* 2021;23(4):e21466.

For peer review only

For peer review only - http://bmjopen.bmj.com/site/about/guidelines.xhtml

Reporting checklist for cross sectional study.

Based on the STROBE cross sectional guidelines.

Instructions to authors

Complete this checklist by entering the page numbers from your manuscript where readers will find each of the items listed below.

Your article may not currently address all the items on the checklist. Please modify your text to include the missing information. If you are certain that an item does not apply, please write "n/a" and provide a short explanation.

Upload your completed checklist as an extra file when you submit to a journal.

In your methods section, say that you used the STROBE cross sectionalreporting guidelines, and cite them as:

von Elm E, Altman DG, Egger M, Pocock SJ, Gotzsche PC, Vandenbroucke JP. The Strengthening the Reporting of Observational Studies in Epidemiology (STROBE) Statement: guidelines for reporting observational studies.

For peer review only

		Reporting Item	Page Number
**Title and abstract**			
Title	#1a	Indicate the study's design with a commonly used term in the title or the abstract	1

Abstract	#1b	Provide in the abstract an informative and balanced summary of what was done and what was found	2
**Introduction**			
Background / rationale	#2	Explain the scientific background and rationale for the investigation being reported	3-4
Objectives	#3	State specific objectives, including any prespecified hypotheses	4
**Methods**			
Study design	#4	Present key elements of study design early in the paper	4-5
Setting	#5	Describe the setting, locations, and relevant dates, including periods of recruitment, exposure, follow-up, and data collection	4-5
Eligibility criteria	#6a	Give the eligibility criteria, and the sources and methods of selection of participants.	4
	#7	Clearly define all outcomes, exposures, predictors, potential confounders, and effect modifiers. Give diagnostic criteria, if applicable	4-5
Data sources / measurement	#8	For each variable of interest give sources of data and details of methods of assessment (measurement). Describe comparability of	4-5

For peer review only

		assessment methods if there is more than one group. Give information separately for for exposed and unexposed groups if applicable.	
Bias	#9	Describe any efforts to address potential sources of bias	n/a we reflect on selection bias and other potential biases in the limitations section
Study size	#10	Explain how the study size was arrived at	5
Quantitative variables	#11	Explain how quantitative variables were handled in the analyses. If applicable, describe which groupings were chosen, and why	5
Statistical methods	#12a	Describe all statistical methods, including those used to control for confounding	5
Statistical methods	#12b	Describe any methods used to examine subgroups and interactions	n/a
Statistical methods	#12c	Explain how missing data were addressed	n/a – no missing data since completion of the survey was only possible after all questions were answered

For peer review only

Statistical methods	#12d	If applicable, describe analytical methods taking account of sampling strategy	n/a
Statistical methods	#12e	Describe any sensitivity analyses	n/a

Results

Participants	#13a	Report numbers of individuals at each stage of study—eg numbers potentially eligible, examined for eligibility, confirmed eligible, included in the study, completing follow-up, and analysed. Give information separately for for exposed and unexposed groups if applicable.	n/a – study was a cross-sectional survey
Participants	#13b	Give reasons for non-participation at each stage	n/a - study was a cross-sectional survey
Participants	#13c	Consider use of a flow diagram	n/a
Descriptive data	#14a	Give characteristics of study participants (eg demographic, clinical, social) and information on exposures and potential confounders. Give information separately for exposed and unexposed groups if applicable.	6-7
Descriptive data	#14b	Indicate number of participants with missing data for each variable of interest	n/a – no missing data since completion of the survey was only possible after all

For peer review only

			questions were answered
Outcome data	#15	Report numbers of outcome events or summary measures. Give information separately for exposed and unexposed groups if applicable.	6-13
Main results	#16a	Give unadjusted estimates and, if applicable, confounder-adjusted estimates and their precision (eg, 95% confidence interval). Make clear which confounders were adjusted for and why they were included	10-13
Main results	#16b	Report category boundaries when continuous variables were categorized	n/a
Main results	#16c	If relevant, consider translating estimates of relative risk into absolute risk for a meaningful time period	n/a
Other analyses	#17	Report other analyses done—e.g., analyses of subgroups and interactions, and sensitivity analyses	n/a
**Discussion**			
Key results	#18	Summarise key results with reference to study objectives	14-16
Limitations	#19	Discuss limitations of the study, taking into account sources of potential bias or imprecision.	15-16

For peer review only

		Discuss both direction and magnitude of any potential bias.	
Interpretation	#20	Give a cautious overall interpretation considering objectives, limitations, multiplicity of analyses, results from similar studies, and other relevant evidence.	16
Generalisability	#21	Discuss the generalisability (external validity) of the study results	14-16
**Other Information**			
Funding	#22	Give the source of funding and the role of the funders for the present study and, if applicable, for the original study on which the present article is based	16

None The STROBE checklist is distributed under the terms of the Creative Commons Attribution License CC-BY. This checklist can be completed online using https://www.goodreports.org/, a tool made by the EQUATOR Network in collaboration with Penelope.ai

For peer review only
